# β-H-Spectrin is a key component of an apical-medial hub of proteins during cell wedging in tube morphogenesis

**DOI:** 10.1242/jcs.261946

**Published:** 2024-08-12

**Authors:** Ghislain Gillard, Katja Röper

**Affiliations:** MRC Laboratory of Molecular Biology, Francis Crick Avenue, Cambridge Biomedical Campus, Cambridge CB2 0QH, UK

**Keywords:** Actomyosin, Microtubules, Cytoskeletal crosslinker, Apical constriction, Morphogenesis, Tubulogenesis, *Drosophila*

## Abstract

Coordinated cell shape changes are a major driver of tissue morphogenesis, with apical constriction of epithelial cells leading to tissue bending. We previously identified that interplay between the apical-medial actomyosin, which drives apical constriction, and the underlying longitudinal microtubule array has a key role during tube budding of salivary glands in the *Drosophila* embryo. At this microtubule–actomyosin interface, a hub of proteins accumulates, and we have shown before that this hub includes the microtubule–actin crosslinker Shot and the microtubule minus-end-binding protein Patronin. Here, we identify two actin-crosslinkers, β-heavy (H)-Spectrin (also known as Karst) and Filamin (also known as Cheerio), and the multi-PDZ-domain protein Big bang as components of the protein hub. We show that tissue-specific degradation of β-H-Spectrin leads to reduction of apical-medial F-actin, Shot, Patronin and Big bang, as well as concomitant defects in apical constriction, but that residual Patronin is still sufficient to assist microtubule reorganisation. We find that, unlike Patronin and Shot, neither β-H-Spectrin nor Big bang require microtubules for their localisation. β-H-Spectrin is instead recruited via binding to apical-medial phosphoinositides, and overexpression of the C-terminal pleckstrin homology domain-containing region of β-H-Spectrin (β-H-33) displaces endogenous β-H-Spectrin and leads to strong morphogenetic defects. This protein hub therefore requires the synergy and coincidence of membrane- and microtubule-associated components for its assembly and function in sustaining apical constriction during tubulogenesis.

## Teaser

**Highlighted Article:** During cellular apical constriction in *Drosophila* tubulogenesis, a protein hub comprising β-H-Spectrin, Shot, Patronin, Filamin and Big bang forms at the interface of apical-medial actomyosin and microtubules.

## INTRODUCTION

During development, tissues arise from simple precursors or primordia through complex shape changes. In many cases the primordia are simple polarised epithelial sheets that undergo in-plane deformations such as convergence and extension as well as out-of-plane bending or budding deformations. At a cellular level, tissue bending can be induced by coordinated epithelial cell shape changes, going from a columnar or cuboidal shape to a wedge shape. An implementation of such cell wedging widespread throughout evolution occurs in the form of the apical constriction of epithelial cells, driven by apical actomyosin. Actomyosin accumulates within epithelial cells near cell–cell contacts, the adherens junctions, located at the apico-lateral side of cells, the so-called junctional pool. However, during morphogenetic changes, epithelial cells also show a prominent apical-medial pool of actomyosin, just underneath the free apical surface, that is highly dynamic and pulsatile in its accumulation and has been shown to play a key role in the apical constriction observed in many tissues (Gillard and Roper, 2020; Martin et al., 2009; Munjal et al., 2015).

Apical-medial actomyosin undergoes cycles of actin network build-up and myosin recruitment, followed by contraction of the network and disassembly (Munjal et al., 2015). Because the actin network is connected to adherens junctions, each pulse of actomyosin contraction can pull on cell junctions and lead to a constriction of the apical surface of the cell, as long as a ratcheting mechanism or clutch stabilises the new smaller apical area. Otherwise, the cycles of actomyosin contraction lead to apical area fluctuations but not shrinkage (Coravos and Martin, 2016; Martin et al., 2009). Pioneering work, especially in *Drosophila*, has identified several upstream regulators of this dynamic system, such as a G-protein-coupled receptor ligand and several of its receptors that trigger a downstream cascade leading to relocation of a RhoGEF followed by Rho1 and Rho kinase activation (Kerridge et al., 2016). This ultimately drives myosin phosphorylation and activation, but also affects actin regulators such as Diaphanous and hence the network the myosin works on (Mason et al., 2013).

**Fig. 1.**
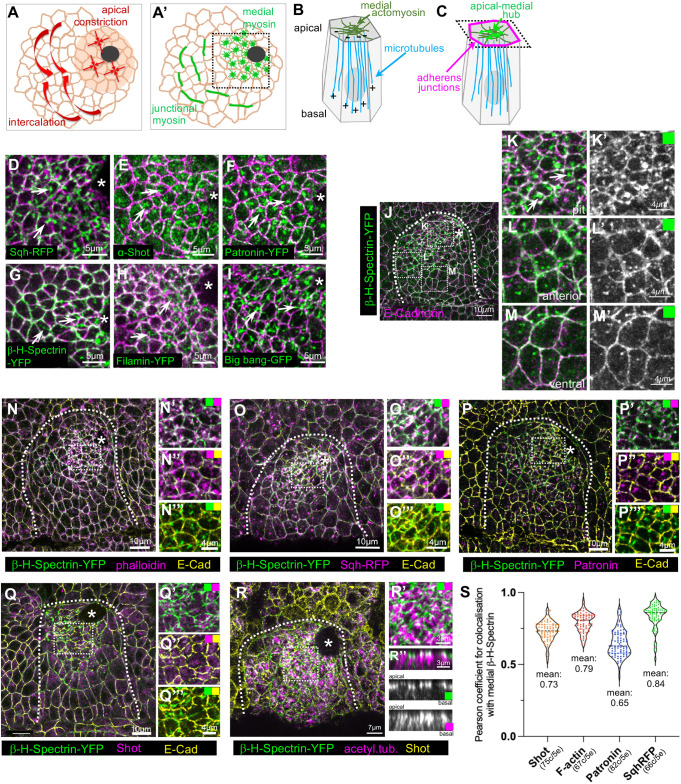
**An apical-medial hub of proteins during apical constriction in tubulogenesis.** (A,A′) Cells in the salivary gland placode during the initial steps of tubulogenesis show two distinct cell behaviours: apical constriction near the forming invagination pit (black circle) and directed intercalations (A). These are driven by distinct pools of apical myosin accumulation: apical-medial actomyosin in the apically constricting cells and polarised junctional accumulations in the cells undergoing intercalations (A′; Sanchez-Corrales et al., 2018, 2021). (B,C) The apical-medial actomyosin is closely juxtaposed to the minus ends of a longitudinal non-centrosomal microtubule array in cells about to apically constrict, and this array and interaction between microtubules and actomyosin is crucial for constriction (Booth et al., 2014; Gillard et al., 2021). (D–I) Proteins localised to the apical-medial position in salivary gland placodal cells about to undergo apical constriction, forming an apical-medial hub (green): (D) myosin II (Sqh–RFP), (E) the spectraplakin Shot, (F) Patronin–YFP, (G) β-H-Spectrin–YFP, (H) Filamin–YFP, (I) Big bang–GFP. Apical membrane outlines are marked by E-Cadherin in magenta. Note that E and F correspond to a triple-labelling for Shot, Patronin–YFP and E-Cadherin. Images are representative of at least 20 analysed placodes. See Fig. S1 for single-channel images of the placodal cells shown in D–I. (J–M′) β-H-Spectrin–YFP (green) is concentrated in the apical-medial position in cells close to the invagination pit, with a lower junctional contribution (J–K′), but is only found at the level of adherens junctions (marked by E-Cadherin, magenta) in non-constricting cells further anterior (J,L,L′) and further towards the ventral midline (J,M,M′). White dotted boxes in J indicate the regions shown in K–M′. Images are representative of at least 20 analysed placodes. (N–R″) Comparison of the apical-medial localisation of various components of the hub. (N–O‴) β-H-Spectrin–YFP (green) colocalises with phalloidin labelling of F-actin (magenta; N–N‴) and with myosin (Sqh–RFP, magenta; O-O‴) in the apical-medial position. (P–Q‴) β-H-Spectrin–YFP (green) also colocalises with Patronin–RFP (magenta, P–P‴) and Shot (magenta, Q–Q‴) in the apical-medial position. (R–R″) β-H-Spectrin–YFP in the apical-medial position (green) colocalises with the ends of the longitudinal microtubule bundles marked by acetylated α-tubulin (acetyl. tub., magenta). Cell outlines are marked by Shot (yellow). (R″) Cross-section view of the cells is shown. Apical membranes in N–Q‴ are labelled by E-Cadherin (E-Cad, yellow). The white dotted boxes in N–R indicate the positions of the higher-magnification two-colour images. Arrows in D–K indicate the apical-medial accumulations. Asterisks in D–R indicate the position of the invagination pit. Dotted lines in J–R mark the boundary of the salivary gland placode. (S) Pearson's correlation coefficient quantification for colocalisation at the apical-medial site of β-H-Spectrin–YFP and either Shot (75 cells from five embryos), Patronin–RFP (82 cells from five embryos), Sqh–RFP (66 cells from five embryos) or F-actin (67 cells from five embryos). Violin plots show the distribution of data points with the median (dashed line) and first and third quartiles (dotted lines) indicated. All images of salivary gland placodes are oriented with anterior side on the left and dorsal side up.

We study the formation of the tubes of the salivary glands in the *Drosophila* embryo as a model process of tube budding from a flat epithelial primordium (Fig. 1A,A′; Fig. S1A,B) (Girdler and Röper, 2014; Sidor and Röper, 2016). We previously identified two key cellular behaviours that in a highly coordinated spatio-temporal pattern drive the initiation of tissue bending as well as the continued invagination of all cells of the primordium (Sanchez-Corrales et al., 2018, 2021). Cells near the invagination point undergo apical constriction driven by strong apical-medial actomyosin accumulation, whereas cells at a distance to the pit undergo convergence and extension in a radially arranged way to continuously feed more cells towards the invagination point. The directional neighbour exchanges in these cells are driven by the polarised accumulation of junctional actomyosin (Fig. 1A,A′). Interestingly, we have shown that the apical-medial pool of actomyosin critically depends on the underlying microtubule cytoskeleton, which in constricting cells becomes organised into a longitudinal array with minus ends anchored apically (Fig. 1B) by the microtubule-interacting proteins Shot and Patronin and the microtubule-severing protein Katanin (Booth et al., 2014; Gillard et al., 2021). If microtubules are depleted or the longitudinal array is not formed, as shown upon Patronin, Katanin or Shot depletion, then apical constriction fails to proceed as in the wild type due to defects in the apical-medial recruitment of actomyosin (Booth et al., 2014; Gillard et al., 2021). Shot, the sole fly spectraplakin protein, is a huge protein with possibilities for many protein interactions. In particular, with an N-terminal actin-binding domain and a C-terminal microtubule-binding domain, Shot is thus able to crosslink the two cytoskeletal systems (Röper et al., 2002). Patronin and Shot have been shown to interact in other tissues as well as in vertebrate cells (Khanal et al., 2016; Nashchekin et al., 2016; Noordstra et al., 2016), and can further interact with Katanin as well as Spectrin (Khanal et al., 2016). A close apposition of apical actomyosin and longitudinal microtubules appears to be conserved across other invagination or bending processes and across evolution, having also been observed in the eye disc morphogenetic furrow *in Drosophila*, as well as in bottle cell constriction during gastrulation and during neural tube closure in *Xenopus* (Corrigall et al., 2007; Fernandes et al., 2014; Lee et al., 2007; Lee and Harland, 2007; Suzuki et al., 2010).

α- and β-Spectrins in most epithelial cells form a submembraneous network that is tied into the cortical actin cytoskeleton. Spectrins usually form heterotetramers of two α and two β subunits that can bind and crosslink membrane adaptors, transmembrane receptors as well as actin filaments directly (Liem, 2016). In epithelial cells, the distribution of α-Spectrin is homogeneous, whereas different β subunits are specific for the apical and basolateral domains. In *Drosophila* two β subunits exist, with β-Spectrin found basolaterally and β-heavy (H)-Spectrin (also known as Karst) restricted to the apical domain (Thomas and Kiehart, 1994). Typically, β-spectrins in vertebrates associate with ankyrin adaptor proteins via a central region and with actin, α-catenins and protein 4.1 (EBP41) via an N-terminal domain, and they also contain a C-terminal pleckstrin homology (PH) domain that can mediate phospholipid binding (Leterrier and Pullarkat, 2022). β-H-Spectrin contains many more spectrin repeats than β-Spectrin but lacks the ankyrin-binding domain; instead, an extended C-terminal domain (β-H-33) appears crucial for membrane association (Williams et al., 2004).

We identified that at the critical microtubule–actomyosin–membrane interface in epithelial cells undergoing apical constriction during tube budding, a large number of proteins accumulate. In addition to Shot and Patronin, which we have previously reported (Gillard et al., 2021), we found that the actin crosslinkers β-H-Spectrin and Filamin (also known as Cheerio) as well as the multi-PDZ-domain adaptor protein Big bang (Bbg; Forest et al., 2018) also accumulate at this interface. We identified β-H-Spectrin as a key component of this apical-medial hub of proteins and as being required for apical-medial F-actin accumulation and apical constriction. In the absence of β-H-Spectrin, Shot and Patronin were reduced in their localisation, but microtubules still became organised into a longitudinal array. However, this usual reorganisation of the microtubule network was not sufficient to trigger proper morphogenesis in embryos depleted of β-H-Spectrin, likely because the apical-medial accumulation of actomyosin and Bbg was also reduced. As opposed to other constituents of the apical-medial hub of proteins, such as Shot and Patronin, the apical-medial accumulation of β-H-Spectrin, as well as that of Bbg, did not rely on microtubules. Rather, overexpression of the C-terminal β-H-33 domain of β-H-Spectrin including the PH domain, but not without it, led to a specific loss of the apical-medial pool of β-H-Spectrin, as well as highly aberrant apical constriction and tube morphogenesis. This suggests that phospholipid binding is key to the localisation of β-H-Spectrin to the apical-medial region. In summary, membrane-associated β-H-Spectrin and its interactors, as well as microtubule minus ends and microtubule minus end-interacting proteins, are required in synergy to support apical-medial actomyosin in its function during cell constriction and tube morphogenesis.

## RESULTS

### A dynamic apical-medial hub of components assembles during apical constriction-driven tube budding

During tube morphogenesis of the salivary glands in the *Drosophila* embryo, the cells that are about to internalise at any given point show a particular arrangement of their cytoskeletal systems: a dense network of apical-medial actomyosin in very close apposition to the minus ends of a longitudinal microtubule array that runs the apical–basal length of the cells (Fig. 1B,C) (Booth et al., 2014; Gillard et al., 2021). In addition to myosin accumulation (Fig. 1D), we have previously found that the spectraplakin Shot (Fig. 1E) and the microtubule minus-end-binding protein Patronin (Fig. 1F) localise within the apical-medial region (Booth et al., 2014; Gillard et al., 2021), using endogenously tagged versions of Patronin (Patronin–YFP; Nashchekin et al., 2016) and the myosin regulatory light chain, Spaghetti squash (Sqh–RFP; Ambrosini et al., 2019) compared to antibody labelling for Shot (Fig. 1E; Fig. S1C–E) (Röper and Brown, 2003). Using further protein trap lines tagging genes at endogenous loci, we uncovered that two actin crosslinkers, β-H-Spectrin (Fig. 1G) (Thomas and Kiehart, 1994) and Filamin A (Fig. 1H) (Sokol and Cooley, 1999) also accumulated in an apical-medial position, as did the multi-PDZ-domain protein Bbg, which has previously been reported to bind Spectrins (Fig. 1I; Fig. S1F–H) (Forest et al., 2018). β-H-Spectrin, like the other components identified here, was strongly enriched at the apical-medial position in cells of the salivary gland placode that were actively constricting apically or about to do so, whereas other placodal and epidermal cells showed a junctional accumulation of β-H-Spectrin (Fig. 1J–M′). To assess whether all these components were in fact localised to the same apical-medial domain, we performed pairwise comparisons between β-H-Spectrin–YFP and the other apical-medial-accumulating proteins. We first co-labelled β-H-Spectrin–YFP and the actomyosin cytoskeleton, using phalloidin to label F-actin (Fig. 1N–N‴), as well as myosin regulatory light chain (Sqh–RFP; Fig. 1O–O‴). Similarly, β-H-Spectrin–YFP localisation was compared with microtubule minus ends, assessed through the endogenous localisation of Patronin (Fig. 1P–P‴) and Shot (Fig. 1Q–Q‴), or by using an antibody against acetylated α-tubulin that accumulates at the apical end of placodal microtubules (Fig. 1R–R″) (Booth et al., 2014). In all cases, quantification of the Pearson's correlation coefficient in fixed samples revealed that all these components colocalised within the same apical-medial foci (Fig. 1S). Thus, in epithelial cells of the salivary gland placode about to undergo or already undergoing apical constriction, a hub of proteins assembles at the interface between the contractile apical-medial actomyosin and the minus ends of the longitudinal microtubule array.

**Fig. 2.**
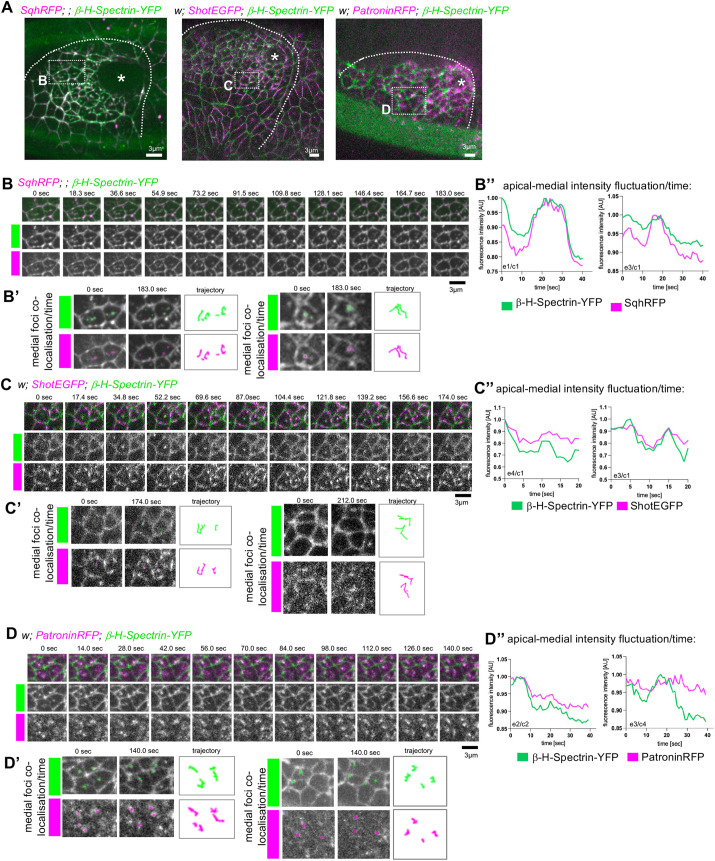
**Proteins of the apical-medial hub show linked dynamics in the apical domain.** (A) Still images of two-colour time-lapse movies of *Sqh–RFP;; β-H-Spectrin–YFP*, of *w; Shot–EGFP; β-H-Spectrin–YFP* and of *w; Patronin–RFP; β-H-Spectrin–YFP* analysed further in higher-magnification views in B–D′. Asterisks mark the position of the invagination pit, dotted lines mark the boundary of the placode and dotted boxes mark the position of the higher-magnification time points shown in B–D. (B) Close-up time-lapse images of individual placodal cells of *Sqh–RFP;; β-H-Spectrin–YFP* embryos, illustrating the colocalisation of apical-medial foci of myosin (Sqh–RFP) and β-H-Spectrin–YFP. (B′) Examples of individual trajectories of apical-medial foci of β-H-Spectrin–YFP and Sqh–RFP over the time period shown in B. (B″) Examples of apical-medial fluorescence intensity fluctuations of β-H-Spectrin–YFP and Sqh–RFP in individual cells [embryo IDs (e) and cell IDs (c) are shown on the plots]. (C) Close-up time-lapse images of individual placodal cells of *w; Shot–EGFP; β-H-Spectrin–YFP* embryos, illustrating the colocalisation of apical-medial foci of β-H-Spectrin–YFP and Shot–EGFP. (C′) Examples of individual trajectories of apical-medial foci of Shot–EGFP and β-H-Spectrin–YFP over the time period shown in C. (C″) Examples of apical-medial fluorescence intensity fluctuations of β-H-Spectrin–YFP and Shot–EGFP in individual cells (embryo and cell IDs shown on plots). (D) Close-up time-lapse images of individual placodal cells of *w; Patronin–RFP; β-H-Spectrin–YFP* embryos, illustrating the colocalisation of apical-medial foci of Patronin–RFP and β-H-Spectrin–YFP. (D′) Examples of individual trajectories of apical-medial foci of Patronin–RFP and β-H-Spectrin–YFP over the time period shown in D. (D″) Examples of apical-medial fluorescence intensity fluctuations of β-H-Spectrin–YFP and Patronin–RFP in individual cells (embryo and cell IDs shown on plots). Data are representative of five movies per genotype. See Fig. S2 for further examples of apical-medial fluorescence intensity fluctuations. AU, arbitrary units. All images of salivary gland placodes are oriented with anterior side on the left and dorsal side up.

Apical-medial myosin driving apical constriction displays a very dynamic behaviour, undergoing pulsatile increases and decreases in intensity as well as flow behaviour underneath the free apical surface (Munjal et al., 2015), and this is, for instance, also mirrored by the behaviour of the apical actin network on which the myosin works (Dehapiot et al., 2020). To investigate whether the components of the apical-medial hub colocalise in time as well as in space, we collected time-lapse movies of β-H-Spectrin–YFP in comparison to Sqh–RFP, Shot–EGFP and Patronin–RFP (Fig. 2A–D″; Fig. S2, Movies 1–3). In all cases, apical-medial β-H-Spectrin–YFP and apical-medial myosin, Shot and Patronin displayed near identical trajectories across the apical surface (Fig. 2B′,C′,D′). When we analysed intensity fluctuations or pulses (as apical-medial intensity over time), β-H-Spectrin–YFP and both Sqh–RFP and Shot–EGFP appeared to fluctuate in intensity in synchrony (Fig. 2B″,C″; Fig. S2A,B). By contrast, Patronin–RFP did not consistently fluctuate in intensity alongside β-H-Spectrin–YFP but remained more steady overall (Fig. 2D″; Fig. S2C). This is likely due to Patronin being bound and stabilising microtubule minus ends directly. These data highlight the formation of a dynamic apical-medial hub that appears to assemble and disassemble similar to myosin as cells undergo apical constriction in the salivary gland placode.

### β-H-Spectrin is required for effective apical constriction and for assembly and maintenance of the apical-medial hub

**Fig. 3.**
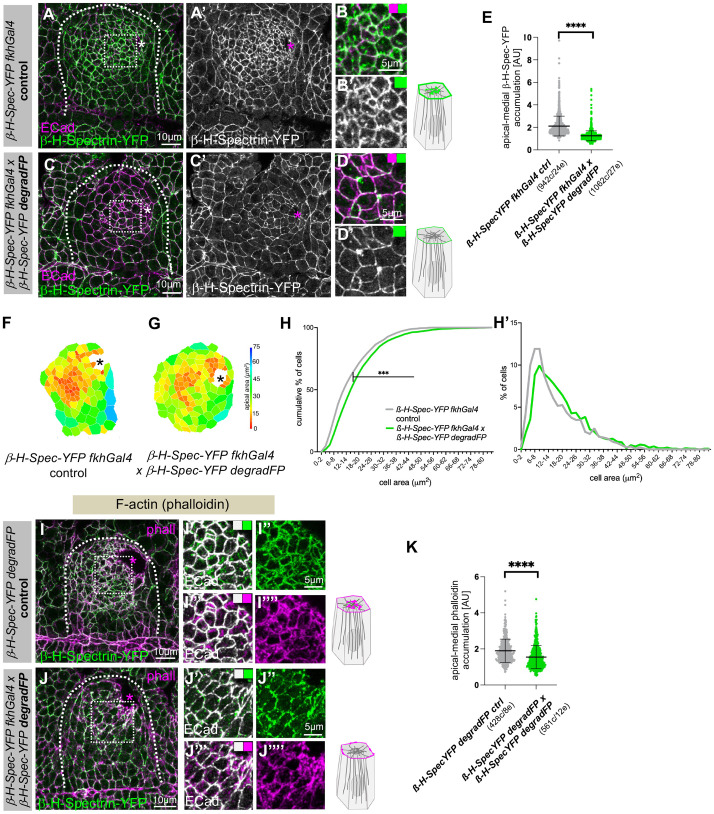
**Loss of β-H-Spectrin impairs apical constriction.** (A–E) In comparison to control placodes (*β-H-Spectrin–YFP fkhGal4*; A–B′), salivary gland tissue-specific degradation of endogenously tagged β-H-Spectrin–YFP (under control of *fkhGal4*) through the expression of an F-box/anti-GFP-nanobody fusion protein, degradFP (*β-H-Spectrin–YFP fkhGal4×β-H-Spectrin–YFP degradFP*) leads to significant loss of β-H-Spectrin–YFP (C–D′), in particular the apical-medial pool. Cell outlines are marked by E-Cadherin (E-Cad, magenta). B,B′ and D,D′ are higher-magnification views of the white dotted boxes marked in A and C, respectively. (E) Quantification of apical-medial β-H-Spectrin–YFP degradation (*β-H-Spectrin–YFP fkhGal4* ctrl, *n*=942 cells from 24 embryos; *β-H-Spectrin–YFP fkhGal4×β-H-Spectrin–YFP degradFP, n*=1062 cells from 27 embryos). Data are presented as mean±s.d. Statistical significance was determined by two-sided unpaired Mann–Whitney test (****P<0.0001). (F–H′) β-H-Spectrin–YFP degradation (G) leads to impairment of apical constriction compared to control (F); apical areas of cells of example placodes are shown as heat maps. (H,H′) Quantification of apical area distribution of placodal cells in control (*β-H-Spectrin–YFP fkhGal4 control*) and β-H-Spectrin-depleted (*β-H-Spectrin–YFP fkhGal4×β-H-Spectrin–YFP degradFP*) placodes, showing the cumulative percentage of cells relative to apical area size (H) and the percentage of cells in different size bins (H′). Statistical significance was determined by Kolmogorov–Smirnov two-sample test (***P<0.001). A total of 12 placodes were segmented and analysed for the control and 15 placodes were segmented and analysed for β-H-Spectrin–YFP depletion. The total number of cells traced was 1319 for control embryos and 1135 for β-H-Spectrin-depleted embryos. (I–K) β-H-Spectrin–YFP degradation (J–J⁗) leads to reduction in apical-medial F-actin (phalloidin, phall; magenta) accumulation compared to that in the control (I–I⁗). β-H-Spectrin–YFP is shown in green and E-Cadherin to label apical cell outlines is shown in white. I′–I⁗ and J′–J⁗ are higher-magnification views of the white dotted boxes marked in I and J, respectively. (K) Quantification of apical-medial phalloidin signal in placodal cells in control (*β-H-Spectrin–YFP degradFP* ctrl, 428 cells from eight embryos) and β-H-Spectrin-depleted (*β-H-Spectrin–YFP fkhGal4×β-H-Spectrin–YFP degradFP*, 561 cells from 12 embryos) placodes. Data are presented as mean±s.d. Statistical significance was determined by two-sided unpaired Mann–Whitney test (****P<0.0001). Asterisks indicate the position of the invagination pit, dotted lines mark the boundary of the salivary gland placode and schematics indicate changes to the analysed component. All images of salivary gland placodes are oriented with anterior side on the left and dorsal side up. AU, arbitrary units.

β-H-Spectrin–YFP showed a very distinct localisation to the apical-medial hub only in those cells about to undergo or already undergoing apical constriction (Fig. 1J–M′), and β-H-Spectrin has previously been shown to be required for some aspects of apical constriction during gastrulation in *Drosophila* embryos (Krueger et al., 2020). We therefore set out to determine whether β-H-Spectrin is required for this cell shape change within the salivary gland placode during tubulogenesis. In order to reduce β-H-Spectrin levels in a tissue-specific manner and not throughout the embryo, we employed the UAS-degradFP system, which targets YFP- or GFP-tagged proteins for degradation by the proteasome (Caussinus et al., 2012; Gillard et al., 2021), in combination with *fkhGal4*, which drives specific expression in the salivary gland placode (Henderson and Andrew, 2000; Zhou et al., 2001). In control embryos (Fig. 3A–B′ and E; *β-H-Spectrin–YFP fkhGal4*), β-H-Spectrin–YFP displayed a junctional pool in all epithelial cells and a strong apical-medial accumulation in cells undergoing apical constriction near the forming invagination pit (Fig. 3B′). When β-H-Spectrin–YFP was targeted for degradation (Fig. 3C–E; *β-H-Spectrin–YFP fkhGal4×UAS-degradFP; β-H-Spectrin–YFP*) the overall level of β-H-Spectrin–YFP was reduced throughout the salivary gland placode where *fkhGal4* was expressed, and whereas some junctional labelling remained, the apical-medial pool of β-H-Spectrin–YFP was strongly reduced (Fig. 3D′,E). This differential response of apical-medial compared to junctional β-H-Spectrin–YFP to degradation could reflect a difference in stability or accessibility due to different spatial arrangements of β-H-Spectrin and associated proteins within both positions. We then analysed the effect this degradation of β-H-Spectrin–YFP had on apical constriction. In comparison to control placodes, where apically constricting cells were clustered near the invagination pit (Fig. 3F), the placodes of β-H-Spectrin–YFP-depleted embryos displayed fewer cells with apically constricted apices (Fig. 3G–H′).

**Fig. 4.**
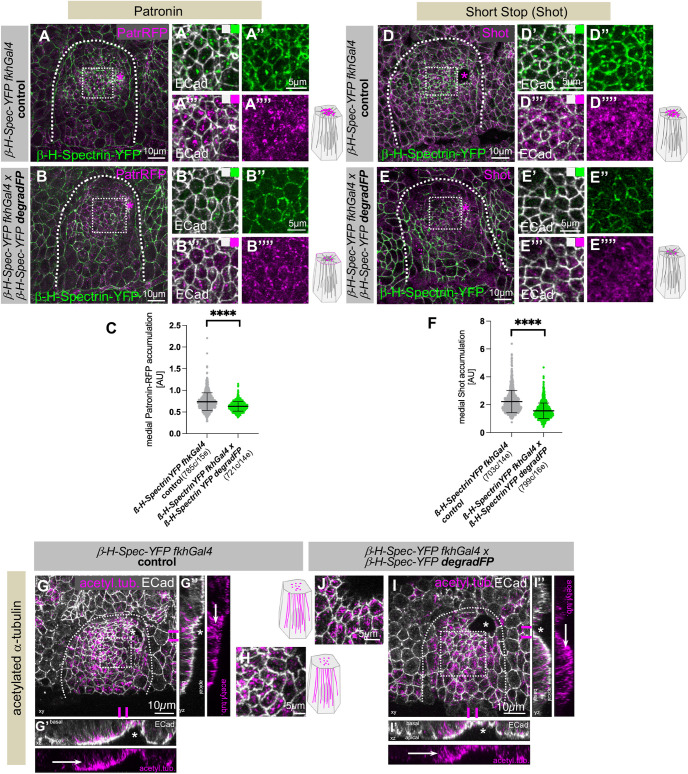
**Loss of β-H-Spectrin leads to loss of the apical-medial hub.** (A–C) β-H-Spectrin–YFP degradation (B–B⁗) leads to a reduction in apical-medial Patronin foci (Patronin–RFP, PatrRFP; magenta) compared to control (A–A⁗). β-H-Spectrin–YFP is shown in green and E-Cadherin (ECad) to label apical cell outlines is shown in white. A′–A⁗ and B′–B⁗ are higher-magnification views of the white dotted boxes marked in A and B, respectively. (C) Quantification of apical-medial Patronin in placodal cells in control (*β-H-Spectrin–YFP fkhGal4* control; 785 cells from 15 embryos) and β-H-Spectrin-depleted (*β-H-Spectrin–YFP fkhGal4×β-H-Spectrin–YFP degradFP*; 721 cells from 14 embryos) placodes. Data are presented as mean±s.d. Statistical significance was determined by two-sided unpaired Mann–Whitney test (****P<0.0001). (D–F) β-H-Spectrin–YFP degradation (E–E⁗) leads to reduction in apical-medial Shot (magenta) foci compared to that in the control (D–D⁗). β-H-Spectrin–YFP is shown in green and E-Cadherin to label apical cell outlines is shown in white. D′–D⁗ and E′–E⁗ are higher-magnification views of the white dotted boxes marked in D and E, respectively. (F) Quantification of apical-medial Shot in placodal cells in control (*β-H-Spectrin–YFP fkhGal4* control; 703 cells from 14 embryos) and β-H-Spectrin-depleted (*β-H-Spectrin–YFP fkhGal4×β-H-Spectrin–YFP degradFP*; 799 cells from 16 embryos) placodes. Data are presented as mean±s.d. Statistical significance was determined by two-sided unpaired Mann–Whitney test (****P<0.0001). (G–J) β-H-Spectrin–YFP degradation (I–J) does not affect formation of longitudinal microtubules (acetylated α-tubulin, acetyl. tub.; magenta), which look comparable to those in the control (G–H). G′,G″ and I′,I″ are cross sections across the invagination pit at the positions indicated by magenta lines in G and I, respectively. White arrows point to longitudinal microtubule bundles. H and J show higher-magnification views of the white dotted boxes marked in G and I, respectively, illustrating microtubule bundle ends visible within the apical domain as illustrated in the schematics. E-Cadherin to label apical cell outlines is in white. Images are representative of at least 20 analysed placodes. See Fig. S3D,E for β-H-Spectrin–YFP images corresponding to G and I, respectively. Asterisks indicate the position of the invagination pit, dotted lines mark the boundary of the salivary gland placode and schematics indicate changes to the analysed component. All images of salivary gland placodes are oriented with anterior side on the left and dorsal side up. AU, arbitrary units.

As β-H-Spectrin is an actin binder and was found to colocalise with apical-medial actomyosin, we analysed F-actin localisation in placodes where β-H-Spectrin was degraded (Fig. 3I–K). In contrast to control placodes, where F-actin displayed a strong medial pool (Fig. 3I–I⁗), when β-H-Spectrin was degraded medial F-actin was also lost, whereas the junctional pool was not affected (Fig. 3J–K). We also analysed the effect of β-H-Spectrin depletion on other components of the apical-medial hub of proteins. Previous work in a different tissue context has shown that β-H-Spectrin can also interact with both Shot and Patronin, two proteins that we previously demonstrated localise to the apical-medial minus ends of microtubules and are key to proper apical constriction in the salivary gland placode (Booth et al., 2014; Gillard et al., 2021). β-H-Spectrin also interacts and collaborates with Bbg in wing discs (Forest et al., 2018). We therefore investigated the effect of β-H-Spectrin depletion on Patronin, Shot and Bbg localisation in placodal cells. In comparison to strong apical-medial foci of Patronin (visualised by endogenously tagged Patronin–RFP) in constricting salivary gland placodal cells of control embryos (Fig. 4A–A⁗), when β-H-Spectrin–YFP was degraded we observed a reduction of apical-medial Patronin foci and an increased localisation at junctions (Fig. 4B–C). For both Shot and Bbg, the apical-medial foci observed under control conditions (Fig. 4D–D⁗; Fig. S3A–A⁗) were also reduced when β-H-Spectrin–YFP was degraded (Fig. 4E–F; Fig. S3B–C).

Thus, loss of β-H-Spectrin leads to concomitant changes in the localisation of other components of the apical-medial hub of proteins. We found previously that Patronin itself is recruited to the apical-medial region of placodal cells by microtubule minus ends, and that when Patronin is lost from these cells, the microtubule cytoskeleton does not form the longitudinal array. We therefore analysed the effect of loss of β-H-Spectrin on the microtubule cytoskeleton. Labelling for acetylated α-tubulin showed that in both control embryos (Fig. 4G–H; Fig. S3D) and embryos with β-H-Spectrin depletion in the placode (Fig. 4I–J; Fig. S3E), microtubules in cells near the invagination point were organised into a longitudinal array with apical foci of microtubule bundles (Fig. 4H,J) and extended bundles visible in cross sections of placodal cells (Fig. 4G′,G″,I′,I″). This suggests that the reduced levels of Patronin we observed in the absence of β-H-Spectrin did not prevent Patronin from performing its role in the reorganisation of the microtubule array. The levels of apical-medial Patronin were still sufficient to capture microtubule minus ends released by Katanin, thereby generating the non-centrosomal microtubules that then form the longitudinal array, as we have shown previously (Gillard et al., 2021). These data illustrate that both the longitudinal microtubule array and apical-medial β-H-Spectrin are required to support apical-medial actomyosin assembly and function to drive apical constriction.

### β-H-Spectrin is recruited to the apical-medial hub independently of microtubules

**Fig. 5.**
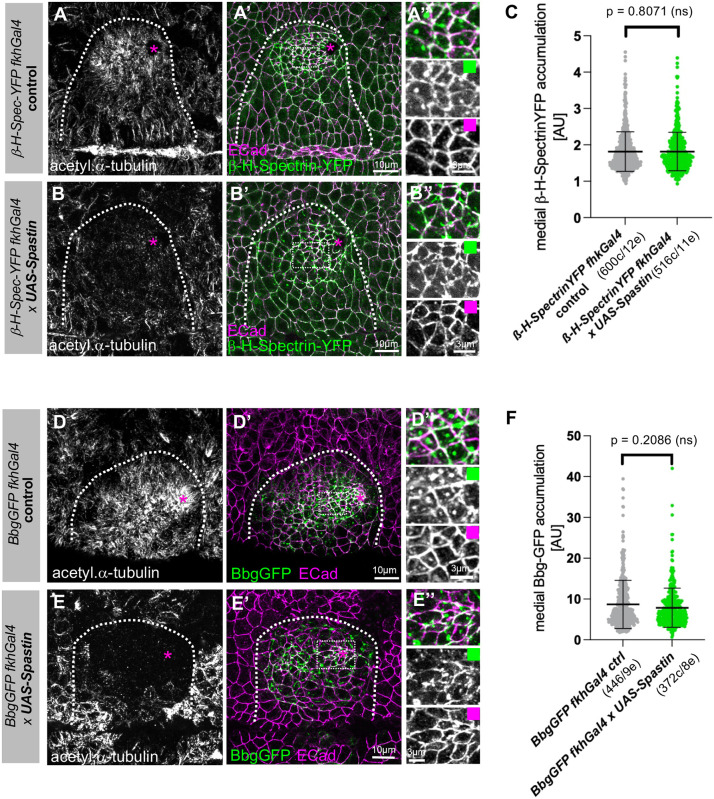
**Microtubules are not required to recruit β-H-Spectrin or Big bang to the apical-medial hub.** (A–C) In contrast to Patronin and Shot, localisation of β-H-Spectrin–YFP to apical-medial foci is not affected when microtubules are lost upon expression of *UAS-Spastin* under *fkhGal4* control (A–A″ for *β-H-Spectrin–YFP fkhGal4* control and B–B″ for *β-H-Spectrin–YFP fkhGal4*×*UAS-Spastin*). A″ and B″ are higher-magnification views of the white dotted boxes marked in A′ and B′, respectively. (C) Quantification of apical-medial β-H-Spectrin–YFP accumulation upon placodal microtubule loss (*β-H-Spectrin–YFP fkhGal4* control, 600 cells from 12 embryos; *β-H-Spectrin–YFP fkhGal4×UAS-Spastin*, 516 cells from 11 embryos). Data are presented as mean±s.d. Statistical significance was determined by two-sided unpaired Mann–Whitney test (*P*=0.8071; ns, not significant). (D–E″) Localisation of Bbg–GFP to apical-medial foci is not affected when microtubules are lost upon expression of *UAS-Spastin* under *fkhGal4* control (D–D″ for *Bbg–GFP fkhGal4* control and E–E″ for *Bbg–GFP fkhGal4×UAS-Spastin*. D″ and E″ are higher-magnification views of the white dotted boxes marked in D′ and E′, respectively. (F) Quantification of apical-medial Bbg–GFP accumulation upon placodal microtubule loss (*Bbg–GFP fkhGal4* control, 446 cells from nine embryos; *Bbg–GFP fkhGal4×UAS-Spastin*, 372 cells from eight embryos). Data are presented as mean±s.d. Statistical significance was determined by two-sided unpaired Mann–Whitney test (*P*=0.2086; ns, not significant). Microtubules are labelled in A,B,D and E with an antibody against acetylated α-tubulin, which accumulates in the longitudinal array in placodal cells. E-Cadherin (ECad) labelling, which marks cell outlines, is shown in magenta. Asterisks indicate the position of the invagination pit and dotted lines mark the boundary of the salivary gland placode. All images of salivary gland placodes are oriented with anterior side on the left and dorsal side up. AU, arbitrary units.

We next turned our attention to how β-H-Spectrin itself is recruited to the apical-medial position. Both Patronin and Shot not only depend on β-H-Spectrin–YFP for their wild-type levels of apical-medial localisation but also depend on the presence of the longitudinal microtubule array in the constricting cells of the salivary gland placode (Booth et al., 2014; Gillard et al., 2021). The formation of the longitudinal microtubule array is in turn crucial for maintenance of the apical-medial pool of actomyosin (Booth et al., 2014). Therefore, in order to assess whether microtubules are required for β-H-Spectrin localisation, we depleted microtubules in the salivary gland placode by overexpressing the microtubule-severing protein Spastin (Fig. 5; expressing *UAS-Spastin* under *fkhGal4* control). In both the control embryos (Fig. 5A–A″,C; *β-H-Spectrin–YFP fkhGal4*) and when microtubules were depleted (Fig. 5B–C; *β-H-Spectrin–YFP fkhGal4×UAS-Spastin*), β-H-Spectrin–YFP was localised to apical-medial positions in apically constricting cells with no significant difference in the amount that accumulated.

Bbg has previously been shown by yeast-two hybrid and co-immunoprecipitation analyses to bind to β-H-Spectrin – an interaction that is necessary for stable localisation of Bbg to adherens junctions in wing discs (Forest et al., 2018). We identified that β-H-Spectrin is also key to robust Bbg localisation to the apical-medial hub in salivary gland placodal cells (Fig. S3). We next tested whether Bbg also depended on an intact microtubule cytoskeleton for its apical-medial localisation in placodal cells or remained apical-medial, bound by β-H-Spectrin, when microtubules were depleted. Like β-H-Spectrin, in both the control (Fig. 5D–D″,F; *Bbg–GFP fkhGal4*) and when microtubules were depleted (Fig. 5E–F; *Bbg–GFP fkhGal4×UAS-Spastin*), GFP-tagged Bbg was localised to apical-medial positions in apically constricting cells with no significant difference in the amount that accumulated.

Thus, it appears that the apical-medial hub has two groups of components that either, like Patronin and Shot, depend on the underlying longitudinal microtubule cytoskeleton for their localisation or that, like β-H-Spectrin and Bbg, are independent of it and therefore are recruited in a different, uncharacterised fashion.

### Phosphoinositide binding by the PH domain of β-H-Spectrin contributes to its recruitment to the apical-medial hub

**Fig. 6.**
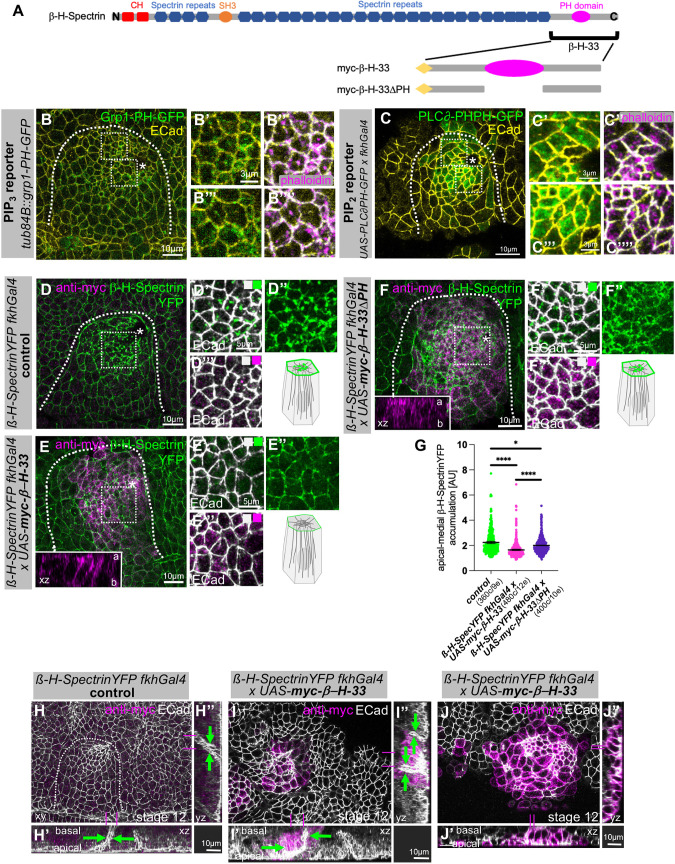
**β-H-Spectrin localisation to and function in the apical–medial hub depends on its PH domain.** (A) Schematic of β-H-Spectrin protein domains, comprising N-terminal calponin homology (CH) domains, 30 Spectrin repeats, an SH3 domain and the β-H-33 C-terminal domain, which contains a phosphoinositide-binding PH domain. Indicated are also the parts of the C-terminal domain contained within overexpression constructs called *UAS-myc-β-H-33* and *UAS-myc-β-H-33*Δ*PH*. (B–C⁗) Analysis of PI(3,4,5)P_3_ and PI(4,5)P_2_ localisation within the salivary gland placode, using the tagged PH domain of Grp1 as a PI(3,4,5)P_3_ reporter (*tub84B::grp1-PH–GFP*) and the PH domain of PLCδ as a PI(4,5)P_2_ reporter (*UAS-PLCδPH–EGFP×fkhGal4*). (B–B⁗) Grp1-PH–GFP is shown in green, phalloidin to label F-actin, including apical-medial pools, is shown in magenta and apical cell outlines marked by E-Cadherin (ECad) are shown in yellow. Areas marked by white dotted boxes in B are magnified in B′,B″ and B‴,B⁗. (C–C⁗) PLCδ-PH–EGFP is shown in green, phalloidin to label F-actin, including apical-medial pools, is shown in magenta and apical cell outlines marked by E-Cadherin are shown in yellow. Areas marked by white dotted boxes in C are magnified in C′,C″ and C‴,C⁗. Images are representative of at least 20 analysed placodes. (D–G) Overexpression of the C-terminal region of β-H-Spectrin, β-H-33, including or lacking the PH domain in the salivary gland placode. Expression of *UAS-myc-β-H-33* (E–E‴) but not *UAS-myc-β-H-33*Δ*PH* (F–F‴), in comparison to *fkhGal4* control (D–D‴), leads to loss of apical-medial β-H-Spectrin–YFP accumulation. Insets in E and F show *xz* cross sections of placodal cells (a, apical; b, basal), illustrating the localisation of myc–β-H-33 (E) and myc–β-H-33ΔPH (F), with myc–β-H-33 localised to free apical and lateral membranes and myc–β-H-33ΔPH mostly cytoplasmic with minimal membrane localisation. Areas marked by white dotted boxes in D, E and F are magnified in D′-D⁗, E′-E⁗, F′-F⁗. β-H-Spectrin–YFP is shown in green, anti-myc signal is shown in magenta and apical cell outlines marked by E-Cadherin are shown in white. (G) Quantification of apical-medial β-H-Spectrin–YFP accumulation upon *UAS-myc-β-H-33* or *UAS-myc-β-H-33*Δ*PH* expression compared to control (*β-H-Spectrin–YFP fkhGal4* control, 360 cells from nine embryos; *β-H-Spectrin–YFP fkhGal4×UAS-myc-β-H-33*, 480 cells from 12 embryos; *β-H-Spectrin–YFP fkhGal4×UAS-myc-β-H-33*Δ*PH*, 400 cells from ten embryos). Data are presented as mean±s.d. Statistical significance was determined by two-sided unpaired Mann–Whitney test (*P<0.01, ****P<0.0001). AU, arbitrary units. In B–E, asterisks indicate the position of the invagination pit and dotted lines mark the boundary of the salivary gland placode. Schematics show the changes to endogenous β-H-Spectrin observed*.* (H–J″) Overexpression of *UAS-myc-β-H-33* using *fkhGal4* at a slightly later stage of tube invagination (embryonic stage 12) shows many poorly constricted cells and only cells with remaining apical-medial β-H-Spectrin–YFP still constricting (I–J″) in comparison to control (H–H″). Anti-myc staining is shown in magenta and apical cell outlines marked by E-Cadherin are shown in white. See Fig. S4 for corresponding β-H-Spectrin–YFP and anti-myc staining images. Cross sections in I′,I″ and J′,J″ illustrate aberrant or delayed invagination of the tube and aberrant or multiple lumen shapes (green arrows) compared to the single narrow lumen in control (green arrows in H′,H″). Magenta lines in H,I,J indicate the position of cross sections. Dotted line in H indicates the boundary of the placode. Placodes in I and J are marked by transgene expression under *fkhGal4* control and anti-myc labelling (magenta). Images are representative of at least 20 analysed placodes. All images of salivary gland placodes are oriented with anterior side on the left and dorsal side up.

What other mechanism could mediate recruitment of β-H-Spectrin to the apical-medial position? The β-H-Spectrin C-terminal domain beyond the spectrin repeats, the β-H-33 fragment, contains a PH domain that can interact with inositol phospholipids (Fig. 6A) (Williams et al., 2004; Zhang et al., 1995). We therefore investigated where phosphoinositides are localised within salivary gland placodal epithelial cells. We employed two transgenic markers of phosphoinositides: Grp1-PH–GFP, which has been reported to bind phosphatidylinositol (3,4,5)-trisphosphate [PI(3,4,5)P_3_] (Fig. 6B–B⁗; *tub84B::grp1-PH–GFP*) (Britton et al., 2002); and PLCδPH–EGFP, which has been reported to bind phosphatidylinositol (4,5)-bisphosphate [PI(4,5)P_2_] (Fig. 6C–C⁗; *fkhGal4×UAS-PLCδPH–EGFP*) (Zelhof and Hardy, 2004). Classically, PI(4,5)P_2_ has been proposed to be important for apical membrane identity, whereas PI(3,4,5)P_3_ appears to be enriched basolaterally in three-dimensional cyst cultures of Madin–Darby canine kidney (MDCK) cells (Martin-Belmonte et al., 2007; Roman-Fernandez et al., 2018). Recent studies during different processes of tissue morphogenesis in *Drosophila* have shown, though, that apical PI(3,4,5)P_3_ can also play an important role (Claret et al., 2014; Miao et al., 2021; Pinal et al., 2006). Ubiquitous expression of Grp1-PH–GFP revealed apical-medial pools of PI(3,4,5)P_3_ in constricting cells in the salivary gland placode, colocalising here with F-actin labelled by phalloidin (Fig. 6B–B⁗). Similarly, placode-specific expression of PLCδPH–EGFP showed labelling of the apical-medial region and hence presence of PI(4,5)P_2_, in part overlapping with F-actin labelling by phalloidin (Fig. 6C–C⁗). Thus, phosphoinositides were concentrated in a position that could support β-H-Spectrin recruitment.

We then aimed to disrupt potential β-H-Spectrin recruitment via phosphoinositides by expressing a construct containing the β-H-33 fragment (Williams et al., 2004). Ectopic expression of this construct has previously been shown to interfere with normal salivary gland development (Williams et al., 2004), but the underlying reason for this has remained unclear. Expression of *UAS-myc-β-H-33* in the salivary gland placode under *fkhGal4* control, in comparison to control (Fig. 6D–D‴,G), led to a reduction of the apical-medial pool of β-H-Spectrin (Fig. 6E–E‴,G). We observed that myc–β-H-33 itself localised to the plasma membrane of placodal cells (see cross section in inset in Fig. 6E), including the free apical domain (Fig. 6E‴). By contrast, expression of a version of β-H-33 lacking the PH domain, *UAS-myc-β-H-33*Δ*PH*, under *fkhGal4* control did not affect the apical-medial pool of β-H-Spectrin (Fig. 6F–G), and this version displayed a much more cytoplasmic distribution (see cross section in inset in Fig. 6F). Where the *myc-β-H-33*Δ*PH* construct localised to the plasma membrane apically it did not colocalise with β-H-Spectrin-YFP (Fig. 6F-F‴). In slightly older salivary gland placodes (stage 12 rather than stage 11 as shown before), cells expressing high levels of myc–β-H-33 showed dilated apices, and only the few cells that retained apical-medial β-H-Spectrin foci appeared contracted (Fig. 6I–J″), in contrast to the normal pattern of graded constriction observed in the control (Fig. 6H–H″; Fig. S4A–C′). Where portions of the placode managed to invaginate in myc–β-H-33-expressing placodes, they formed an irregular-shaped tube (Fig. 6I′,I″).

These data suggest that β-H-Spectrin is, at least in part, recruited to the apical-medial region of placodal cells via its C-terminal PH domain, through interactions with phosphoinositides. We show that perturbation of this interaction through expression of a dominant-negative interactor, in the form of β-H-33, leads to aberrant apical constriction and aberrant tubulogenesis.

### DISCUSSION

The submembraneous spectrin cytoskeleton has been studied in many different contexts since spectrins were originally identified as core components of the red blood cell cytoskeleton. In many instances, including in red blood cells but also (as more recently identified) in axons, a key role for spectrins is membrane or cell stabilisation. This role is possible due to the expandable and hence reversibly stretchable structure of spectrin repeats. In both red blood cells and axons, the spectrin heterotetramers, together with associated proteins such as membrane receptors and actin filaments, are arranged in highly regular assemblies (Leterrier and Pullarkat, 2022; Teliska and Rasband, 2021). In epithelial cells, by contrast, two β-Spectrin subunits are expressed and show a polarised distribution, with β-H-Spectrin confined to the apical domain and β-Spectrin localised basolaterally, as well as a less regular arrangement. Epithelial spectrins have been shown to play roles in epithelial morphogenesis in different organisms, including embryo elongation in *Caenorhabditis elegans* (Jia et al., 2019; McKeown et al., 1998), eye and follicle cell morphogenesis in *Drosophila* (Lee et al., 2010; Ng et al., 2016), and more recently also mesoderm invagination (Krueger et al., 2020). This requirement in dynamic developmental processes suggests that spectrins also serve important functions beyond stabilising and buffering a fixed cell shape.

**Fig. 7.**
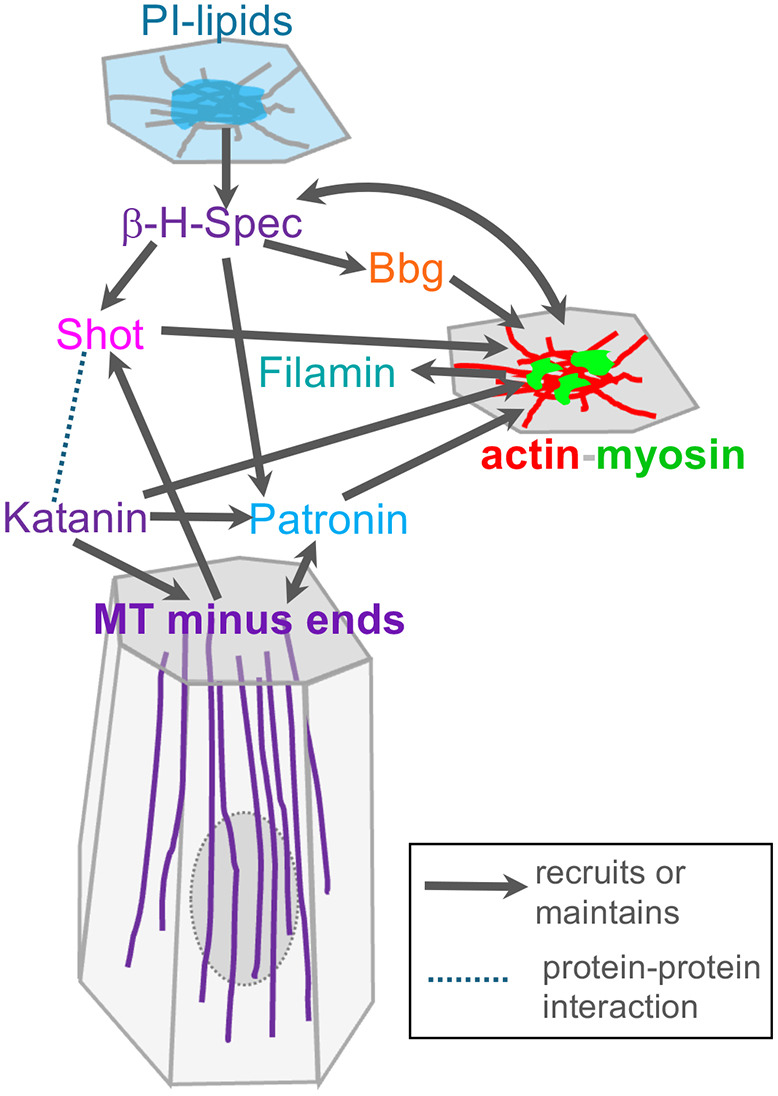
**The apical-medial hub of cytoskeletal interactors.** Schematic of the components identified that localise at the interface of apical-medial actomyosin and microtubule minus ends in placodal cells. The schematic illustrates which components recruit or maintain other components, or otherwise interact with them (where requirement for recruitment is not yet clear). β-H-Spectrin is central to many interactions. MT, microtubule; PI, phosphoinositide.

We provide evidence for a very dynamic function of β-H-Spectrin in the rapid apical constriction of cells about to invaginate to form a tube, during the morphogenesis of the salivary glands in the fly embryo. Such apical constriction is driven by a highly dynamic and pulsatile pool of actomyosin within the apical-medial domain of cells, and β-H-Spectrin not only colocalises with but also dynamically co-pulsates with myosin and other components of this apical-medial hub. A related function has previously been proposed for β-H-Spectrin in mesoderm invagination in the fly embryo, although is not required for apical constriction per se but rather for apical ratcheting that allows the stabilisation of the cell apical domain after a pulse of actomyosin-driven constriction (Krueger et al., 2020). We identify that β-H-Spectrin colocalises not only with actin and myosin at the apical-medial site, but also with the microtubule minus-end-binding protein Patronin and the cytolinker Shot, as well as with Filamin and the scaffold protein Bbg, thus forming an apical-medial hub of interacting proteins at the interface of actomyosin and microtubules (Fig. 7). We show that β−H-Spectrin depletion reduces the apical-medial accumulation of Patronin and Shot. All three proteins have in fact been shown to directly interact in *Drosophila* follicle cells, with β-H-Spectrin acting as an upstream factor to recruit both Shot and Patronin at the apical membrane (Khanal et al., 2016). Shot and Patronin are required in the salivary gland placode to promote the formation of non-centrosomal microtubules, the anchoring of their minus ends at the apical membrane and their orientation along the apical-basal axis. This non-centrosomal microtubule network is involved in actomyosin accumulation at the apical-medial site, which is a prerequisite for efficient apical constriction and tissue invagination (Booth et al., 2014; Gillard et al., 2021). The combined function of the hub of proteins at the apical-medial site is as yet unclear, as all of the proteins individually appear to be important for apical constriction, but β-H-Spectrin and Filamin can crosslink actin filaments, Shot can crosslink actin and microtubules, and the other components can bind either actin or microtubules and also other components of the hub, possibly generating a highly interlinked structure (Fig. 7). Whilst this could suggest a more static function, the fact that all components appear to be highly dynamic and follow the pulsatile behaviour of actomyosin suggests that the presence of the hub might allow recruitment of further factors with roles in the regulation of the dynamicity of apical-medial actomyosin. Furthermore, recent modelling approaches have revealed that a certain amount of crosslinking of actin is key for a contractile and pulsatile actomyosin network (Belmonte et al., 2017). Therefore, components of the hub could directly influence the pulsations and contraction of apical-medial actomyosin in the placodal cells.

As we have shown previously, some components of the hub, such as actomyosin, Patronin and Shot, depend on the microtubule cytoskeleton, or rather the apical minus ends of the longitudinal microtubule bundles, for their recruitment or maintenance (Booth et al., 2014; Gillard et al., 2021). By contrast, the apical-medial localisation of β-H-Spectrin and Bbg does not depend on microtubules. For β-H-Spectrin, this localisation rather appears to rely on its C-terminal β-H-33 segment. The β-H-33 segment has in fact previously been shown to exhibit autonomous membrane association in late-stage embryonic salivary glands, in part mediated by the PH domain contained within it (Williams et al., 2004). PH domains are known to interact with phosphoinositides, including PI(4,5)P_2_ and PI(3,4,5)P_3_, and recent findings suggest roles for both PI(4,5)P_2_ and PI(3,4,5)P_3_ within the apical domain of epithelial cells in tissues undergoing morphogenesis in *Drosophila* (Claret et al., 2014; Miao et al., 2021; Pinal et al., 2006). We found that fluorescent probes reporting on the presence of both PI(4,5)P_2_ and PI(3,4,5)P_3_ label the apical-medial domain of constricting salivary gland placodal cells, suggesting that β-H-Spectrin might be recruited to the apical-medial domain via interactions through the PH domain. Furthermore, the fact that overexpression of the full β-H-33 fragment competes with endogenous β-H-Spectrin localisation at the apical-medial hub, leading to major morphogenetic defects, whereas overexpression of the β-H-33 segment without its PH domain does not impair the apical-medial accumulation of endogenous β-H-Spectrin, also strongly supports this notion. We propose that Bbg is then recruited through a direct interaction with β-H-Spectrin (Forest et al., 2018). Thus, the hub overall seems to depend on recruitment signals and synergy from two directions: the plasma membrane and the minus ends of longitudinal microtubule bundles. Interestingly, even though Patronin and Shot are reduced in their apical-medial pool when β-H-Spectrin is depleted, Patronin function remains enough to support the formation of the longitudinal array, illustrating that in the wild type, both the longitudinal array as well as β-H-Spectrin and its interactors are key to apical constriction. It will be interesting to address whether in other instances where microtubules and apical actomyosin are closely apposed during apical constriction, and where microtubules are required for the constriction process, a similar hub of proteins is required (Corrigall et al., 2007; Fernandes et al., 2014; Lee et al., 2007; Lee and Harland, 2007; Suzuki et al., 2010).

A further role suggested for β-H-Spectrin might also indicate an additional functionality of the hub as a whole: β-H-Spectrin has been shown to be involved in mechanotransduction, by regulating the Hippo pathway (Fletcher et al., 2015) or endothelial mechano-responses (Mylvaganam et al., 2022). With pulsatile contractile cycles of actomyosin activity during apical constriction clearly exerting forces within a cell and via junctional contacts to neighbouring cells, β-H-Spectrin might also participate in mechanical responses of cells within the placode that help coordinate cell behaviours. Thus, we suspect that many important roles for the spectrin cytoskeleton remain to be uncovered, with our analysis of β-H-Spectrin function within the apical-medial hub illustrating one dynamic role.

## MATERIALS AND METHODS

### *Drosophila* stocks and genetics

The following fly stocks from Bloomington Stock Centre were used in this study: *Daughterless-Gal4* (*Da-Gal4*; #27608); Bbg–GFP (y[1] w[*];; Mi{PT-GFSTF.1}bbg[MI02662-GFSTF.1]/TM6C, Sb[1] Tb[1]) (#60187); and *w[1118]; PBac{602.P.SVS-1}cherCPTI[000847]* (#115123). Additional fly stocks used included: *β-H-Spectrin–YFP/Kst–YFP* (*w1118; PBac{681.P.FSVS-1}kstCPTI002266*) from Kyoto Stock Centre; *Patronin::TagRFPattp40[22H02-C]* (Takeda et al., 2018); *Sqh–TagRFPt[9B]* (Ambrosini et al., 2019); *fkhGal4* on chromosome III (Henderson and Andrew, 2000); *Patronin–YFP* (*w1118; Patronin–YFP/Cyo*) (Nashchekin et al., 2016); *UAS-deGradFP* (*w; If/Cyo; UAS>NSlmb-vhhGFP4/TM6b*) (Caussinus et al., 2012); *UAS-Spastin* on X (Sherwood et al., 2004); *P{w+mc kstβH33.Scer\UAS.T:Hsap\MYC=pβH33}* (Williams et al., 2004); *P{w+mc kstβH33ΔPH.Scer\UAS.T:Hsap\MYC=pβH33ΔPH}* (Williams et al., 2004); *tub84B::grp1-PH–GFP* (Britton et al., 2002); and *UAS-PLCδPH–EGFP* (Zelhof and Hardy, 2004).

The following strains of transgene combinations were generated in the context of this study: *Patronin–RFP; β-H-Spectrin-YFP*, *Sqh–RFP;; β-H-Spectrin–YFP*, *Shot–EGFP; β-H-Spectrin–YFP, β-H-Spectrin–YFP fkhGal4*, *Bbg–GFP fkhGal4*, *UAS-Spastin;; β-H-Spectrin–YFP*, *UAS-Spastin;; Bbg–GFP*, *Patronin–RFP; β-H-Spectrin–YFP fkhGal4* and *UAS-degradFP; β-H-Spectrin–YFP*. Genotypes analysed are indicated in the figure panels and legends and are listed in Table S1.

### Embryo immunofluorescence labelling, confocal microscopy and live analysis

Embryos were collected on apple juice-agar plates and processed for immunofluorescence using standard procedures as below. Briefly, embryos were dechorionated in 50% bleach, fixed in 4% methanol-free formaldehyde, and devitellinised in a 50% mix of 90% ethanol and heptane or of methanol and heptane. They were then stained with phalloidin or with primary and secondary antibodies in PBS plus 0.5% bovine serum albumin and 0.3% Triton X-100. The anti-E-Cadherin (DCAD2, 1:10 dilution) and anti-CrebA (CrebA Rbt-PC, 1:1000 dilution) antibodies were obtained from the Developmental Studies Hybridoma Bank at the University of Iowa. The other primary antibodies used were anti-acetylated α-tubulin (clone 6-11B-1, 1:500 dilution; Sigma), anti-Shot (1:1000 dilution; Röper and Brown, 2003) and anti-myc primary (9E10, 1:500 dilution; Abcam). The following secondary antibodies from Jackson ImmunoResearch Laboratories were used at 1:200: Alexa Fluor 488 AffiniPure donkey anti-rabbit IgG (H+L) (711-545-152); Cy3 AffiniPure donkey anti-rabbit IgG (H+L) (711-165-152); Alexa Fluor 647 AffiniPure donkey anti-rabbit IgG (H+L) (711-605-152); Cy3 AffiniPure donkey anti-mouse IgG (H+L) (715-165-151); Cy5 AffiniPure donkey anti-mouse IgG (H+L) (715-175-151); Cy3 AffiniPure goat anti-rat IgG (H+L) (112-165-167); Alexa Fluor 647 AffiniPure donkey anti-rat IgG (H+L) (712-605-153) and Cy5 AffiniPure donkey anti-guinea pig IgG (H+L) (706-175-148). The following secondary antibodies from Invitrogen were used at 1:200: donkey anti-rabbit IgG (H+L) highly cross-adsorbed secondary antibody, Alexa Fluor Plus 405 (A48258); goat anti-mouse IgG (H+L) highly cross-adsorbed secondary antibody, Alexa Fluor 350 (A-21049); donkey anti-mouse IgG (H+L) highly cross-adsorbed secondary antibody, Alexa Fluor Plus 488 (A32766) and goat anti-rat IgG (H+L) cross-adsorbed secondary antibody, Alexa Fluor 488 (A-11006). Rhodamine–phalloidin (1:500) was from Thermo Fisher Scientific (R415). Samples were embedded in Vectashield (Vectorlabs, H-1000).

Images of fixed samples were acquired on an Olympus FluoView 1200 (with the FV10-ASW v04.02 software) with a 40× objective or on a Leica SP8 inverted microscope (LAS X software) with a 40× objective that was equipped with a 405 nm laser line for four-colour imaging as *z*-stacks to cover the whole apical surface of cells in the placode. *Z*-stack projections and orthogonal sections were assembled in ImageJ (NIH, Bethesda, MD, USA) or Imaris (Bitplane), three-dimensional rendering was performed in Imaris.

For live time-lapse imaging, the embryos were dechorionated in 50% bleach, rinsed in water and attached to a coverslip with the ventral side up using heptane glue (TESA 4510 mixed with heptane) and covered with Halocarbon Oil 27 (Sigma). Time-lapse sequences of embryos of the genotypes *Sqh–RFP;;β-H-Spectrin–YFP* and *Patronin–RFP;; β-H-Spectrin–YFP* were acquired every 6.1 s and 17.4 s, respectively, on a spinning-disc setup (Nikon W1 spinning disc and 40× objective) as *z*-stacks, whereas embryos of the genotype *Shot–EGFP; β-H-Spectrin–YFP* were imaged every 7 s on a Zeiss 880 inverted microscope (Zen 2.3 SP1 FP3 v14.0.20.201 software) with a 40×/1.3NA oil objective as a single confocal slice, using linear unmixing to allow imaging of both fluorophores. *Z*-stack projections to generate movies were assembled in ImageJ or Imaris.

### Quantifications

#### Cell segmentation and apical area analysis

For the analysis of apical cell area, images of fixed embryos of late stage 11 or early stage 12 placodes, with E-Cadherin (also known as Shotgun) labelled to highlight cell membranes and with CrebA labelled to mark salivary gland fate, were analysed. Cells were segmented in confocal image stacks, with cell analysis software (otracks, custom software written in IDL, from L3 Harris Geospatial, https://www.nv5geospatialsoftware.com/Products/IDL; otracks code available on request from Dr Guy Blanchard, gb288@cam.ac.uk) as used and published previously (Blanchard et al., 2009, 2010; Booth et al., 2014; Butler et al., 2009). Briefly, the shape of the curved placode surface was identified in each *z*-stack as a contiguous ‘blanket’ spread over the cortical signal. Quasi-two-dimensional images for cell segmentation containing clear cell cortices were extracted as a maximum intensity projection of the 1 µm or 1.5 µm thick layer of tissue below the blanket. These images were segmented using an adaptive watershed algorithm. Manual correction was used to perfect cell outlines for fixed embryos. Only cells of the salivary placode were used in subsequent analyses and were distinguished based on CrebA staining.

#### Medial accumulation of phalloidin, Bbg–GFP, β-H-Spectrin–YFP, Patronin–RFP and Shot

The analysis of medial accumulation of different components upon β-H-Spectrin depletion was performed on fixed embryos. For phalloidin, Bbg and Shot analyses, staining was performed on *β-H-Spectrin–YFP* embryos, whereas for Patronin analyses, *Patronin–RFP; β-H-Spectrin–YFP* embryos were used. The role of microtubules for β-H-Spectrin–YFP and Bbg–GFP apical-medial accumulation was assessed in embryos of the genotypes *UAS-Spastin; β-H-Spectrin–YFP* and *UAS-Spastin; Bbg–GFP*, respectively. Images were taken of salivary gland placodes and surrounding tissue at late stage 11 or early stage 12. Maximum intensity projections of the apical surface of placodal cells were generated using three to five optical sections separated by 1 µm each in the *z* direction. For each embryo analysed, fluorescence measurements were made for all secretory cells within the placode except cells close to the actomyosin cable. Cell outlines were drawn based on E-cadherin or β-H-Spectrin–YFP signal while salivary gland placodes were identified thanks to the placode-specific CrebA marker or the actomyosin cable and aligned boundary junctions formed around the placode. The medial and junctional mean intensity values were measured after drawing the cell outlines (7-pixel-wide line) with a home-made plugin in Fiji (https://fiji.sc/), which is available on request. Ten cells were similarly analysed in the surrounding tissue.

For β-H-Spectrin–YFP, Bbg–GFP, Shot and phalloidin quantifications, the graphs display the medial accumulation corresponding to the ratio between the medial intensity for each cell in the placode divided by the mean intensity of the ten cells outside the placode. For Patronin–RFP, due to the noisy labelling in the surrounding epidermis, the graph displays the ratio between Patronin–RFP medial staining versus junctional staining for each cell in the placode.

#### Colocalisation analysis

Colocalisation of phalloidin, Sqh–RFP, Shot and Patronin–RFP with β-H-Spectrin–YFP was automatically quantified by calculating the Pearson's correlation coefficient using the JACoP plugin in Fiji (Bolte and Cordelieres, 2006). In total, 75 cells (Shot), 67 cells (phalloidin), 82 cells (Patronin–RFP) and 66 cells Sqh–RFP) exhibiting β-H-Spectrin–YFP apical-medial accumulation were manually segmented and quantified from five different embryos for each genotype. Colocalisation was assessed in flies expressing Sqh–RFP or Patronin–RFP together with β-H-Spectrin–YFP, or in β-H-Spectrin–YFP flies that were stained for Shot or labelled using phalloidin.

### Statistics and reproducibility

Significance was determined using unpaired two-tailed Student's *t*-tests, non-parametric two-tailed Mann–Whitney tests for non-Gaussian distributions, unpaired two-tailed *t*-tests with Welch's correction for data with unequal standard deviations, or Kolmogorov–Smirnov tests for the comparison of cumulative distributions. Tests used are indicated in the figure legends.

## Supplementary Material



10.1242/joces.261946_sup1 Supplementary information
