# Supplementary material for: β-H-Spectrin is a key component of an apical-medial hub of proteins during cell wedging in tube morphogenesis
Source: J Cell Sci. 2024 Aug 12;137(15):jcs261946. doi: 10.1242/jcs.261946 (PMC11361641; doi:10.1242/jcs.261946)
Supplement: Supplementary information [file joces-137-261946-s1.pdf]

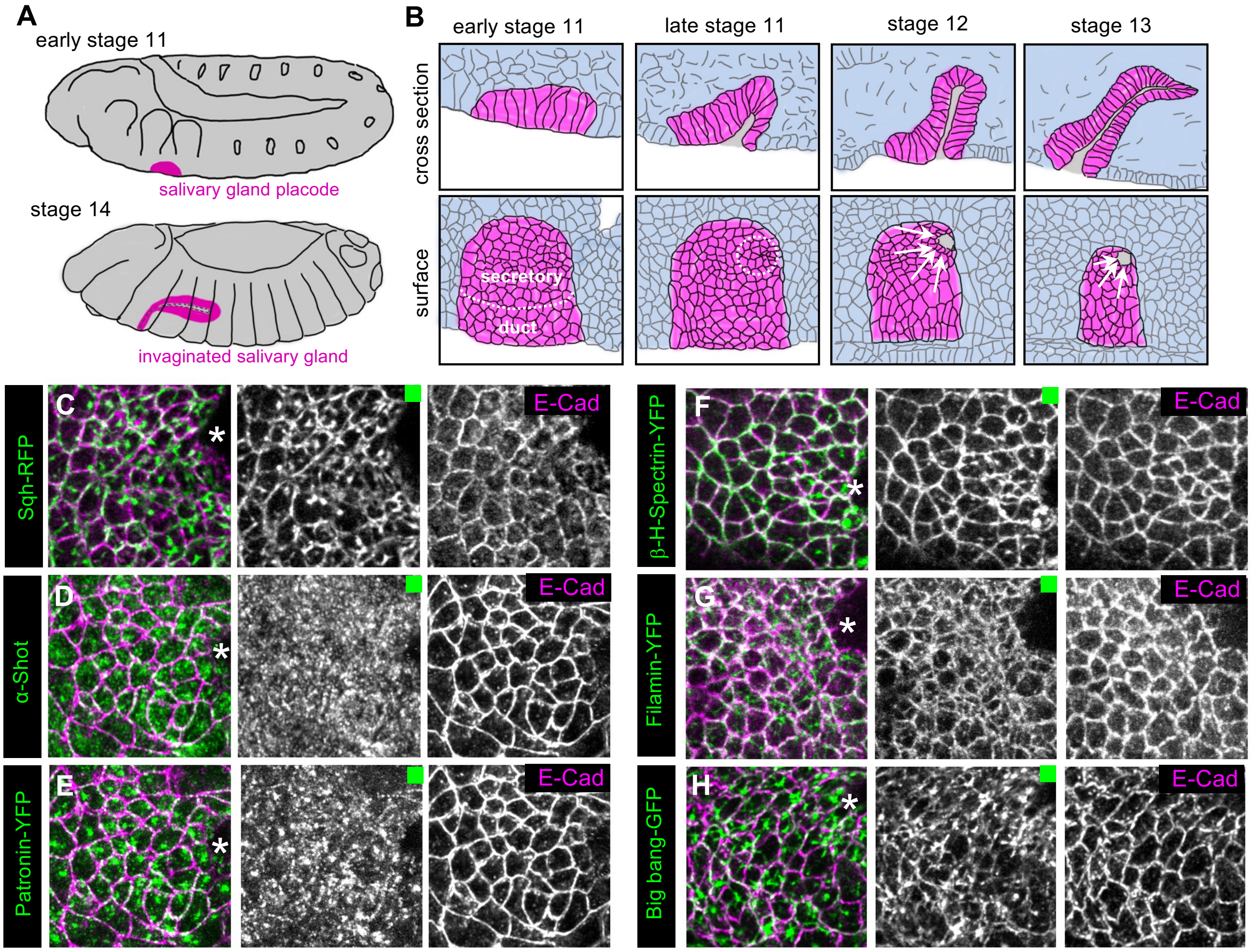

Fig. S1, related to Fig. 1. An apical-medial hub of proteins during apical constriction in tubulogenesis.

A Schematic of *Drosophila* embryos at early stage 11 and stage 14 illustrating the position of the salivary gland placode at early stage 11 (magenta) and invaginated salivary gland at stage 14 (magenta), respectively.

B Cross section and surface view schematics of salivary gland placodal cells (magenta) during the invagination process. Secretory cells begin to constrict in the dorsal posterior corner of the placode (dotted white circle) with cells continuously moving towards the pit through cell intercalations and to then apically constrict and invaginate (white arrows).

C-H Individual channel panels corresponding to the panels shown in Figure 1 D-I. **C** Sqh-RFP, **D** anti-Shot, **E** Patronin-YFP, **F** β-H-Spectrin-YFP, **G** Filamin-YFP, **H** Bbg-GFP, all in green. Cell outlines are labelled for E-Cadherin (magenta). Asterisks indicate the position of the invagination point. Note that **D** and **E** correspond to a triple-labeling for Shot, Patronin-YFP and E-Cadherin.

Journal of Cell Science • Supplementary information

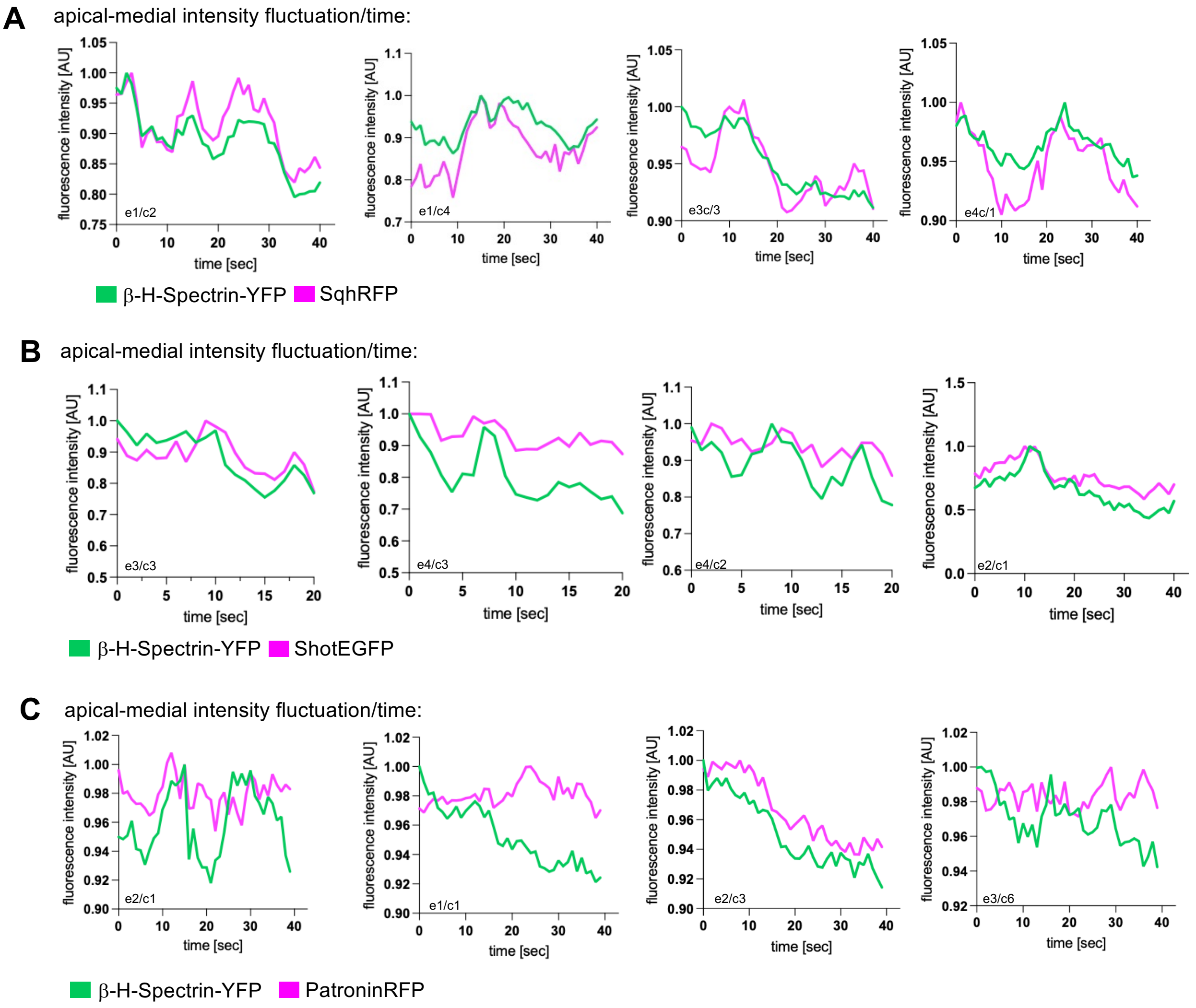

Fig. S2, related to Fig. 2. Proteins of the apical-medial hub show linked dynamics in the apical domain.

A Further examples of apical-medial fluorescence intensity fluctuations of β-H-Spectrin-YFP and SqhRFP in individual cells (embryo and cell IDs shown on plots).

B Examples of apical-medial fluorescence intensity fluctuations of β-H-Spectrin-YFP and Shot-EGFP in individual cells (embryo and cell IDs shown on plots).

C Examples of apical-medial fluorescence intensity fluctuations of β-H-Spectrin-YFP and PatroninRFP in individual cells (embryo and cell IDs shown on plots).

Journal of Cell Science • Supplementary information

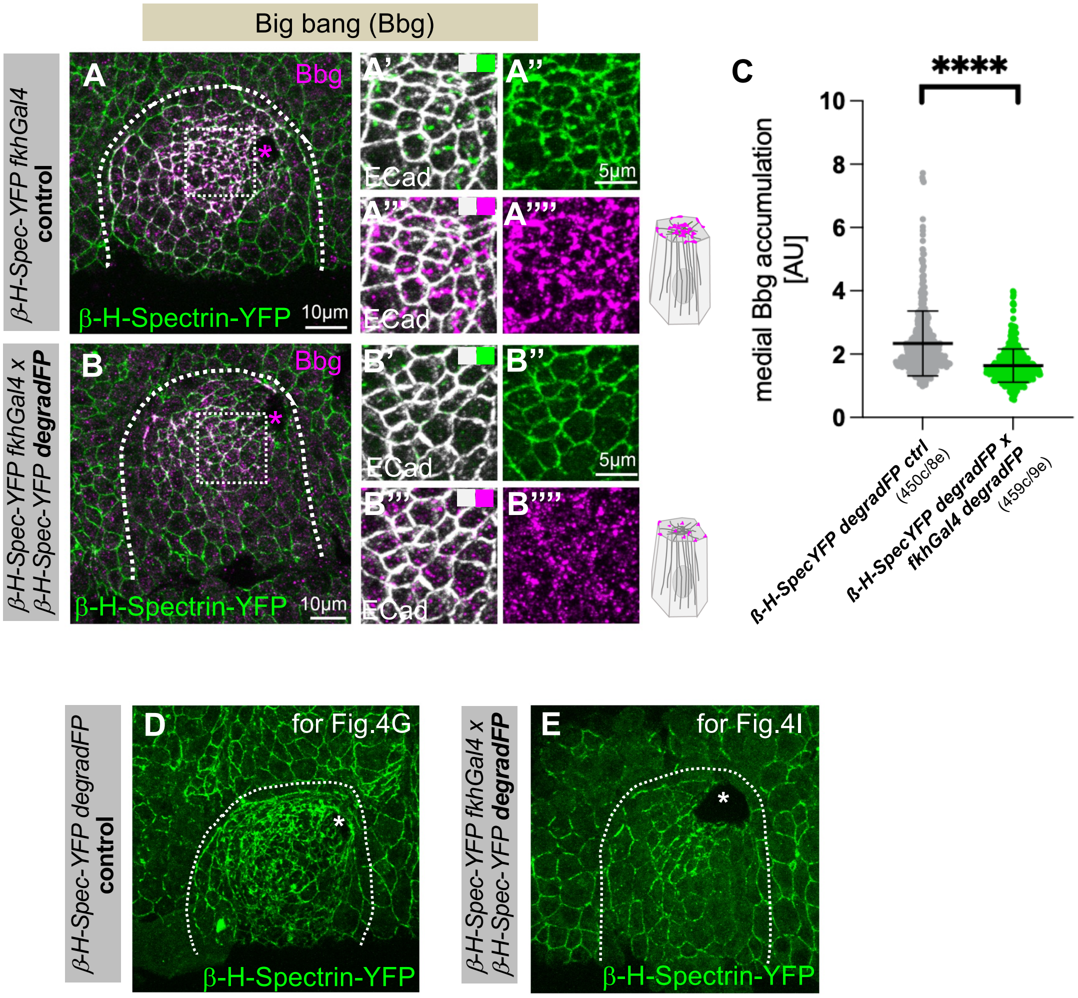

Fig. S3, related to Fig. 4. Loss of β-H-Spectrin leads to loss of the apical-medial hub

A-C β-H-Spectrin-YFP degradation (**B-B''''**) leads to a reduction in apical-medial Bbg foci (anti-Bbg, magenta) compared to control (**A-A''''**). β-H-Spectrin-YFP is in green and E-Cadherin to label apical cell outlines is in white. **A'-A''''** and **B'-B''''** are higher magnifications of the white boxes marked in **A** and **B**, respectively. **C** Quantification of apical-medial Bbg in placodal cells in control (*β-H-Spectrin-YFP fkhGal4 control;* 450 cells from 8 embryos) and β-H-Spectrin depleted (*β-H-Spectrin-YFP fkhGal4* x*β-H-Spectrin-YFP degradFP;* 459 cells from 9 embryos) placodes. Shown are mean +/-SD, statistical significance was determined by two-sided unpaired Mann-Whitney test as p<0.0001.

D,E β-H-Spectrin-YFP in control ((*β-H-Spectrin-YFP fkhGal4;* **D**) and when degraded ((*β-H-Spectrin-YFP fkhGal4* x*β-H-Spectrin-YFP degradFP*; **E**) for the BBg analysis shown in Fig.4 **G-J**.

Asterisks indicate the position of the invagination pit; dashed lines mark the boundary of the salivary gland placode.

Journal of Cell Science • Supplementary information

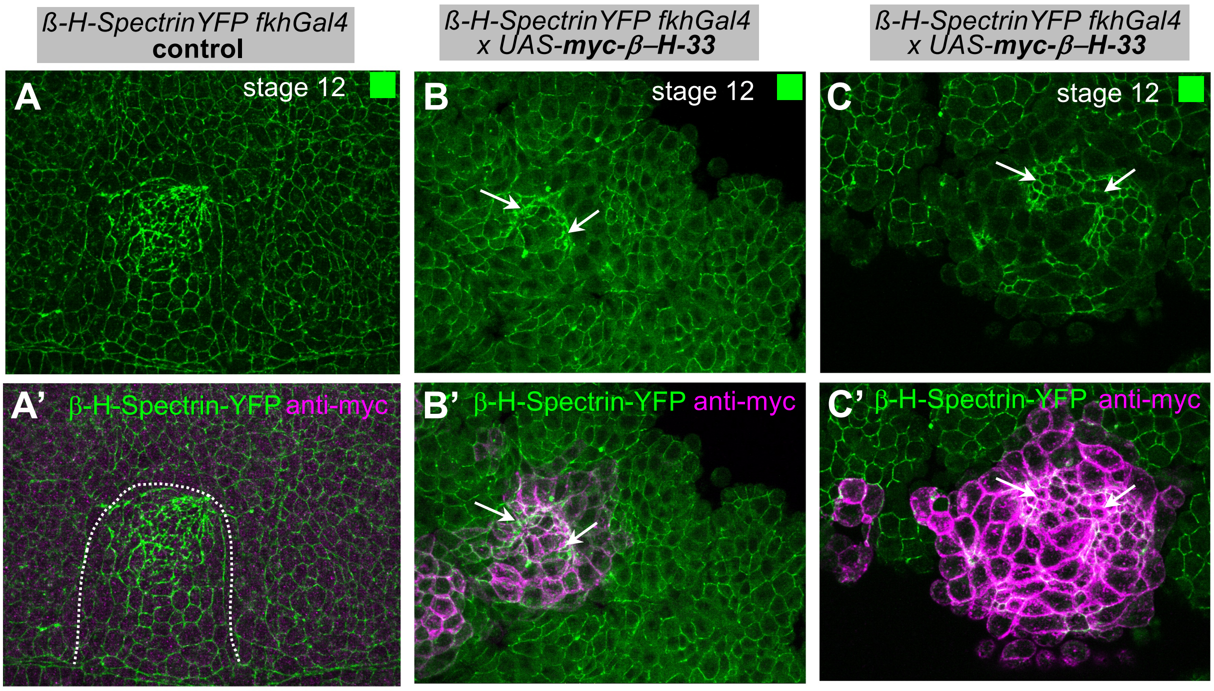

Fig. S4, related to Fig. 6. β-H-Spectrin's localization to and function in the apical-medial hub depends on its PH domain.

A-C' Overexpression of *UAS-myc-β-H-33* using *fkhGal4* at embryonic stage 12 in comparison to control as shown in Figure 6 H, I, J. Shown here are the β-H-Spectrin channel (**A-C**, green) and the overlay of β-H-Spectrin (**A'-C'**, green) and anti-myc staining (**A'-C'**, mangenta) to reveal the overexpressed fragment. Arrows point to the remaining constricting cells when βH33 is overexpressed, the dotted line in **A'** marks the boundary of the placode, in **B'** and **C'** the anti-myc labelling indicates placodal cells due to the use of *fkhGal4*.

Table S1. Genotypes relating to figure panels

Figure panel	Genotype
Fig. 1D	*SqhRFP*
Fig.1 E, F	*Patronin-YFP*
Fig.1 G, J-M’, N-N’’’, Q-Q’’’, R-R’’’	*β-H-Spectrin-YFP*
Fig.1 H	*Cheerio-YFP*
Fig. 1 I	*Bbg-GFP*
Fig. 1 O-O’’’	*Sqh-RFP; ; β-H-Spectrin-YFP*
Fig. 1 P-P’’’	Patronin-RFP; *β-H-Spectrin-YFP*
Fig. 2 A	*Sqh-RFP; ; β-H-Spectrin-YFP*<br>*Shot-EGFP; β-H-Spectrin-YFP*<br>*Patronin-RFP; β-H-Spectrin-YFP*
Fig. 2 B-B’’’	*Sqh-RFP; ; β-H-Spectrin-YFP*
Fig. 2 C-C’’’	*Shot-EGFP; β-H-Spectrin-YFP*
Fig. 2 D-D’’’	*Patronin-RFP; β-H-Spectrin-YFP*
Fig. 3 A-B’, F, I-I’’’’	*β -H-Spectrin-YFP fkhGal4*
Fig. 3 C-D’, G, J-J’’’’	*β-H-Spectrin-YFP fkhGal4*<br>*x β-H-Spectrin-YFP degradFP*
Fig. 4 A-A’’’’	*Patronin-RFP; β-H-Spectrin-YFP fkhGal4*
Fig. 4 B-B’’’’	*Patronin-RFP; β-H-Spectrin-YFP fkhGal4*<br>*x β-H-Spectrin-YFP degradFP*
Fig. 4 D-D’’’’, G-H	*β -H-Spectrin-YFP fkhGal4*
Fig. 4 E-E’’’’, J-I’’	*β-H-Spectrin-YFP fkhGal4*<br>*x β-H-Spectrin-YFP degradFP*
Fig. 5 A-A’’	*β-H-Spectrin-YFP fkh-Gal4*
Fig. 5 B-B’’	*β-H-Spectrin-YFP fkh-Gal4*<br>*x UAS-Spastin; ; β-H-Spectrin-YFP*
Fig. 5 D-D’’	*Bbg-GFP fkhGal4*
Fig. 5 E-E’’	*Bbg-GFP fkhGal4*<br>*x UAS-Spastin; ; Bbg-GFP fkhGal4*
Fig. 6 B-B’’’’	*tub84B::grp1-PH-GFP x fkhGal4*
Fig. 6C-C’’’’	*UAS-PLC∂PH-EGFP x fkhGal4*
Fig. 6 D-D’’’, H-H’’	*β-H-Spectrin-YFP fkh-Gal4*
Fig. 6 E-E’’’, I-J’’	*β-H-Spectrin-YFP fkh-Gal4*<br>*x UAS-myc-β-H-33*
Fig. 6 F-F’’’	*β-H-Spectrin-YFP fkh-Gal4*<br>*x UAS-myc-β-H-33ΔPH*
Suppl. Fig. S1C	*SqhRFP*
Suppl. Fig. S1 D, E	*Patronin-YFP*
Suppl. Fig. S1 F	*β-H-Spectrin-YFP*
Suppl. Fig. S1 G	*Cheerio-YFP*

Journal of Cell Science • Supplementary information

Suppl. Fig. S1 H	*Bbg-GFP*
Suppl. Fig. S2A	*Sqh-RFP; ; β -H-Spectrin-YFP*
Suppl. Fig. S2B	*Shot-EGFP; β-H-Spectrin-YFP*
Suppl. Fig. S2C	*PatroninRFP; β-H-Spectrin-YFP*
Suppl. Fig. S3 A-A'''', D	*β -H-Spectrin-YFP fkhGal4*
Suppl. Fig. S3 B-B'''', E	*β-H-Spectrin-YFP fkhGal4*<br>*x β-H-Spectrin-YFP degradFP*
Suppl. Fig. S4 A-A'	*β -H-Spectrin-YFP fkhGal4*
Suppl. Fig. S4 B-C'	*β-H-Spectrin-YFP fkh-Gal4 x UAS-myc-β-H-33*

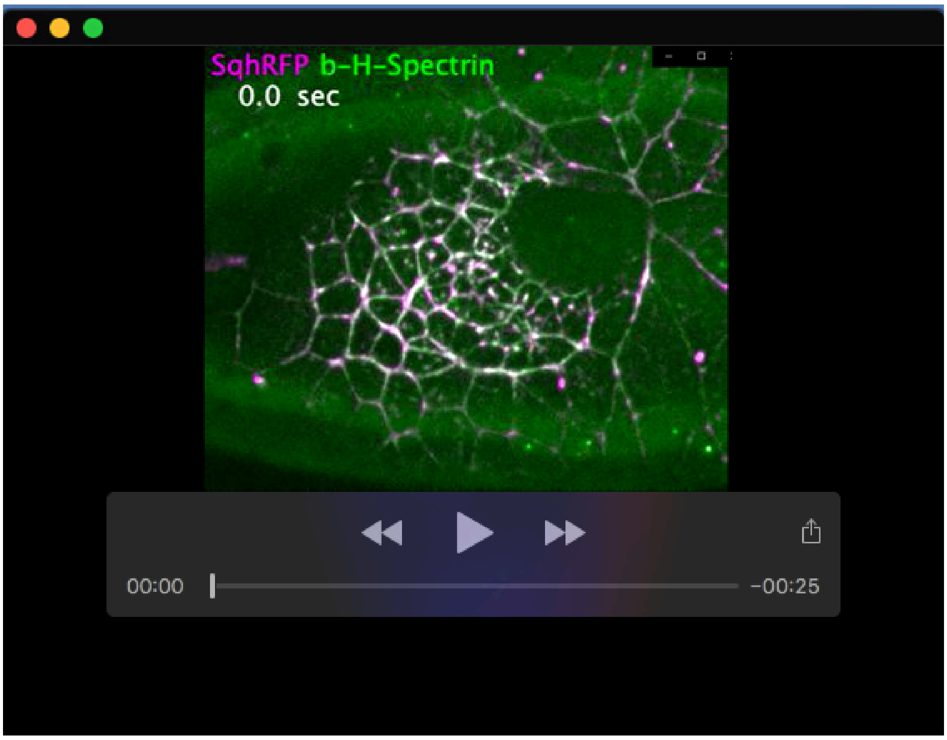

Movie 1. Dynamic behaviour of myosin and β-H-Spectrin in placodal cells.
Time lapse movie of a salivary gland placode of an embryo with the genotype *Sqh-RFP; β- H-Spectrin-YFP*, with Sqh-RFP in magenta and β-H-Spectrin-YFP in green. Time interval is indicated.

Journal of Cell Science • Supplementary information

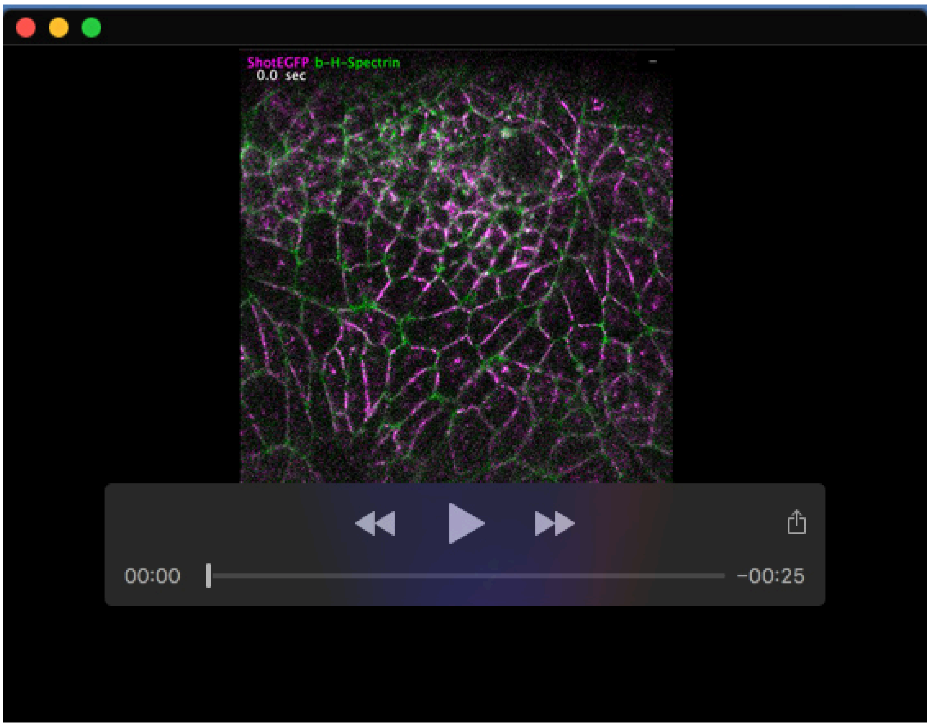

Movie 2. Dynamic behaviour of Shot and β-H-Spectrin in placodal cells. Time lapse movie of a salivary gland placode of an embryo with the genotype *Shot-EGFP; β-H-Spectrin-YFP*, with Shot-EGFP in magenta and β-H-Spectrin-YFP in green. Time interval is indicated.

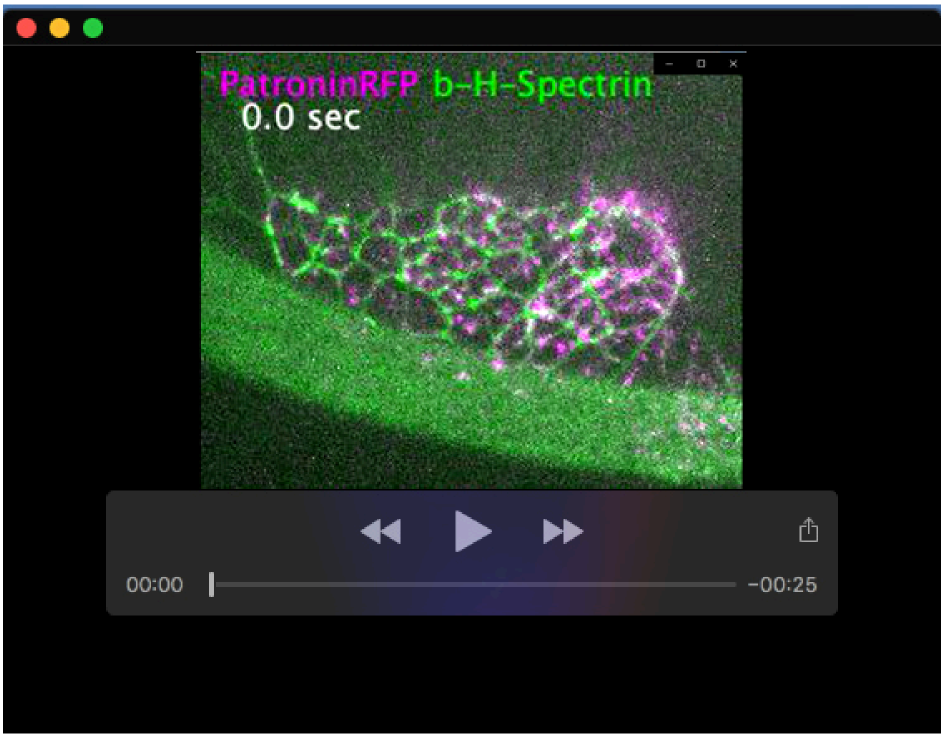

Movie 3. Dynamic behaviour of Patronin and β-H-Spectrin in placodal cells. Time lapse movie of a salivary gland placode of an embryo with the genotype *Patronin-RFP; β-H-Spectrin-YFP*, with Patronin-RFP in magenta and β-H-Spectrin-YFP in green. Time interval is indicated.

Journal of Cell Science • Supplementary information
