# Supplementary material for: β-H-Spectrin is a key component of an apical-medial hub of proteins during cell wedging in tube morphogenesis
Source: J Cell Sci. 2024 Aug 12;137(15):jcs261946. doi: 10.1242/jcs.261946 (PMC11361641; doi:10.1242/jcs.261946)
Supplement: Supplementary file 1 [file joces-137-261946_review_history.pdf]

Journal of Cell Science

ß-H-Spectrin is a key component of an apical-medial hub of proteins during cell wedging in tube morphogenesis

Ghislain Gillard and Katja Röper
DOI: 10.1242/jcs.261946

Editor: Andrew Ewald

Review timeline

Original submission:	8 January 2024
Editorial decision:	27 February 2024
First revision received:	3 June 2024
Accepted:	1 July 2024

Original submission

First decision letter

MS ID#: JOCES/2024/261946

MS TITLE: ß-H-Spectrin is a key component of an apical-medial hub of proteins during cell wedging in tube morphogenesis

AUTHORS: Ghislain Gillard and Katja Röper

We have now reached a decision on the above manuscript.

To see the reviewers' reports and a copy of this decision letter, please go to: https://submit-jcs.biologists.org and click on the 'Manuscripts with Decisions' queue in the Author Area. (Corresponding author only has access to reviews.)

As you will see, the reviewers share enthusiasm for the study and raise a number of substantial criticisms that prevent me from accepting the paper at this stage. They suggest, however, that a revised version might prove acceptable, if you can address their concerns. If you think that you can deal satisfactorily with the criticisms on revision, I would be pleased to see a revised manuscript. We would then return it to the reviewers.

Please ensure that you clearly highlight all changes made in the revised manuscript. Please avoid using 'Tracked changes' in Word files as these are lost in PDF conversion.

I should be grateful if you would also provide a point-by-point response detailing how you have dealt with the points raised by the reviewers in the 'Response to Reviewers' box. Please attend to all of the reviewers' comments. If you do not agree with any of their criticisms or suggestions please explain clearly why this is so.

Reviewer 1

Advance summary and potential significance to field

This manuscript by Gillard and Röper explores the molecular components of an important but poorly understood cellular domain, the apical cortex of epithelial cells. Recent work has highlighted the importance of this domain in tissue morphogenesis and signaling processes in

© 2024. Published by The Company of Biologists under the terms of the Creative Commons Attribution License (https://creativecommons.org/licenses/by/4.0/).

developing epithelia, and this work contributes to the understanding of its components and how they interact. Additionally, the work provides significant new insights into the role of beta-H-spectrin in organizing this domain.

This is a well-written and for the most part carefully documented manuscript. Although some of its findings have been reported previously by the Röper lab, this work adds significant new information, particularly regarding the role of beta-H-spectrin in organizing the apical medial cortex, and provides synthesis that previously has been lacking. Overall, there are some minor concerns that can be addressed with minimal further analysis and/or alterations to the text, but this work will have impact on the field and will serve as a foundation for future studies.

Comments for the author

Figure 1: Colocalization in the medial domain. The authors argue that beta-H-spectrin colocalizes with a number of other proteins in the apical medial domain. The images presented are reasonably clear, but the assertion would be much stronger if the degree of colocalization was quantified and statistically analyzed over a reasonable number of samples, rather than qualitatively shown in just a single representative photo.

Figure 2: Dynamics of colocalization. Here some quantification is shown graphically, but no statistical analysis (correlation) is presented. Additionally, the rationale for how line scans were positioned (random vs. chosen by some criteria) and which time points were used is not described. Additionally, the movies are very short and of low resolution. As a result, the statements in the second paragraph of the discussion indicating that the work shows highly dynamic behavior by beta-H-spectrin do not seem well founded. Addition of kymographs showing correlated movement over time between different proteins would better support this claim.

Reviewer 2

Advance summary and potential significance to field

This paper reports on studies on the regulation of tubular structure formation during morphogenesis using salivary gland development in Drosophila as a model system. In this system, locally orchestrated apical constriction in a defined region drives the invagination of epithelial cells and initiates tub formation. Apical constriction is a key mechanism of morphogenesis and studying its regulation is important. Using genetics and immunohistochemsity, quantitative live imaging and perturbations such as induced protein degradation and the use of dominant negative tools, the study reports the existence of a hub of proteins at the apical medial area of epithelial cells primed to invaginate. The authors identify ß-H-Spectrin as a key component of this hub, that when acutely degraded leads to defects in apical constriction in the salivary gland context. The study further identifies that some components of the apical medial hub rely on microtubules for their localisation, while some including ß-H-Spectrin and Big Bang do not. For ß-H-Spectrin the authors gather evidence that its ability to interact with phospholipids of the apical plasma membrane perhaps enriched with P(3,4,5)P3 helps to position it at the apical medial area. Therefore, this study provides novel insights into apical constriction regulation in this system with mechanistic insights into protein positioning and function.

The study is carried out overall at high standard. It brings novel mechanistic insights and even clarifies a long-standing observation why overexpressing spectin's PH domain causes dominant negative effects during salivary gland development.
The manuscript could be improved however it its presentation and other aspects listed below before publication.

Comments for the author

1) The abstract could be rewritten to make a clearer distinction between what was known and what is new.

© 2024. Published by The Company of Biologists under the terms of the Creative Commons Attribution License (https://creativecommons.org/licenses/by/4.0/).

2) The model presented in figure 7 is difficult to digest and should be revised. I enjoyed the insightful discussion. Illustrating conceptually what ß-H-Spectrin might do could be better use for this figure.
3) It would be good to discuss why ß-H-Spectrin at the junctions resits degradation, are there different pools with different functions?
4) The change of the apical area certainly also involves contractions at the junctions. How efficient is degradation of ß-H-Spectrin? Its levels at the junctions appear reduced as well. Is the tension in the junctions the same? The lack of apical constriction could be caused by effects on both apical medial and junctional contractility.
5) Figure 2, while I am inclined to follow the authors interpretation, could be more rigorously quantified.
Could you analyse correlations, or better cross correlation and plot those? The areas chosen for line profiles seem a little arbitrary.
6) Figure 3 E clarify the sample size annotation.
7) Figure 3, F, G, example placodes, where is the invagination pit? The white "bay" top rightish? Does this mean there are more cells with reduced apical area around the pit when Spectrin is degraded?
8) E.g. Figure 5. I might have misunderstood, but according to the methods quantifications as in Fig 4C require salivary gland fate visualisation via dCrebA. Both Cadherin and dCrebA are mouse? Clarify legends and quantification basis.
9) Figure 4, it would be a good idea to check that degradFP expression does not affect apical cell area by itself, that control is missing.
10) Parts of the discussion are too redundant (e.g. page 13 second paragraph lower part) with wording in the results.
11) A table with the genotypes for each figure could help and the figure legends could be improved to contain all necessary detail.

Reviewer 3

Advance summary and potential significance to field

This well-written and thorough paper is a welcome addition to the literature on the cytoskeletal basis for apical constriction, a crucial cell shape change that drives epithelial bending during development of many organs and organisms.

Building on the lab's own previous work using the Drosophila salivary gland, the authors describe several new protein components of the so-called "apical hub" that organizes the medial pool of actomyosin that is crucial for apical constriction. The core finding is that B-h-spectrin is present in the hub where it is essential for effective apical constriction. Loss of spectrin leads to loss of several hub components related to actin but does not perturb the organization of microtubules. A final set of structure/function analyses reveals that the spectrin PH domain is essential for its function here and accordingly that local control of phospholipids may be important for apical constriction.

Overall the paper is very convincing and I support publication after a few minor concerns are addressed.

Comments for the author

Comments/Concerns:

1. The section heading on page 7 states flatly that Spectrin "is require for apical constriction and assembly" of the hub, but all the effects shown in Fig 3 are fairly modest. I think it would be more accurate to say that spectrin is required for "effective" or "complete" apical constriction, etc. Hedging here a little bit would not weaken the paper.

2. Two things in Fig 5A'' and B'' don't make sense to me. First, in the images shown, the apical hub as labeled by spectrin in green seems much larger in all cells expressing spastin, but the graph says there's no difference. The graph says "accumulation, so is this just mean pixel intensity? If so, it

© 2024. Published by The Company of Biologists under the terms of the Creative Commons Attribution License (https://creativecommons.org/licenses/by/4.0/).

may not detect a change in hub size. Perhap the authors should quantify area of the hub? Or perhaps they just picked a non-representative image to show int eh figure. Either way this should be resolved.

Second, to my eye, the spastin expression also causes a robust ectopic accumulation of spectrin at the cell cortex. Is this the case? If so it should be quantified and sicsussed. Given the interplay of apical and medial actin populations, this could be an informative result.

3. No arrows lead to or from Filamin in the right-hand panel of Fig. 7.

4. The paper is quite scholarly, with a robust introduction and discussion.
But an opportunity is missed in these sections' very heavy focus on Drosophila and occasionally C. elegans.
It would be useful to the community, I feel, for the authors to mention that microtubules, spectrins, and PH domains are all also implicated in apical constriction by studies in Xenopus.
For example, parallel microtubule arrays with minus ends at the apical surface are a hallmark of apical constriction in Xenopus gastrulation (PMID: 17868669) and neurulation (PMID: 20534674; PMID: 17329357), and Shroom family proteins that control these microtubule arrays and have also been related to spectrin localization (PMID: 19554350). Finally, the PH-domain protein Plekhg5 controls apical constriction in Xenopus gastrulation (PMID: 30446627). These evolutionarily conserved aspects of apical constriction are important especially because so many key regulators in Drosophila apical constriction (e.g. Fog pathway) seem largely not to be.

First revision

Author response to reviewers' comments

Response to Reviewers' Comments

Please find below our detailed responses to all the reviewers' comments and concerns, our responses are in *blue italics*.

Reviewer 1 Advance Summary and Potential Significance to Field:
This manuscript by Gillard and Röper explores the molecular components of an important but poorly understood cellular domain, the apical cortex of epithelial cells. Recent work has highlighted the importance of this domain in tissue morphogenesis and signaling processes in developing epithelia, and this work contributes to the understanding of its components and how they interact. Additionally, the work provides significant new insights into the role of beta-H-spectrin in organizing this domain.

This is a well-written and for the most part carefully documented manuscript. Although some of its findings have been reported previously by the Röper lab, this work adds significant new information, particularly regarding the role of beta-H-spectrin in organizing the apical medial cortex, and provides synthesis that previously has been lacking. Overall, there are some minor concerns that can be addressed with minimal further analysis and/or alterations to the text, but this work will have impact on the field and will serve as a foundation for future studies.

Reviewer 1 Comments for the Author:
Figure 1: Colocalization in the medial domain. The authors argue that beta-H-spectrin colocalizes with a number of other proteins in the apical medial domain. The images presented are reasonably clear, but the assertion would be much stronger if the degree of colocalization was quantified and statistically analyzed over a reasonable number of samples, rather than qualitatively shown in just a single representative photo.

We agree that quantification makes sense for this analysis and have now added a graph in Figure 1S that shows the quantitative analysis (colocalistion analysis for medial b-H-Spectrin compared to Shot, phalloidin, Patronin and myosin), displayed as Pearson coefficients for individual cells (see also new section in Methods).

© 2024. Published by The Company of Biologists under the terms of the Creative Commons Attribution License (https://creativecommons.org/licenses/by/4.0/).

Figure 2: Dynamics of colocalization. Here some quantification is shown graphically, but no statistical analysis (correlation) is presented. Additionally, the rationale for how line scans were positioned (random vs. chosen by some criteria) and which time points were used is not described. Additionally, the movies are very short and of low resolution. As a result, the statements in the second paragraph of the discussion indicating that the work shows highly dynamic behavior by beta-H-spectrin do not seem well founded. Addition of kymographs showing correlated movement over time between different proteins would better support this claim.

We have followed the reviewer's suggestion and analysed the dynamic behaviour in more detail. It is important though to point out some differences between the apical constriction during the process of salivary gland tube formation and for instance mesoderm invagination earlier during gastrulation, a process where this type of analysis has been done in the past. In the mesoderm, all cells begin apical constriction at about the same time, and even though they do not pulse synchronously, in fact neighbours are often anti-coupled, all cells actively constrict as the whole process is very fast (10-15 min). The apical constriction in salivary gland tubulogenesis, as we have shown, occurs over a longer period of time, and also spreads out radially over time from an initially constricting group at the position of the future invagination point to more cells at a distance to the pit (Booth et al., 2014; Sanchez-Corrales et al., 2018 and 2021). Cells that are not actively constricting yet, though, are already building up myosin pulsations (all epidermal cells in fact show low level pulses), but the cycle length of these pulsation cannot yet lead to successful constriction and instead leads to area fluctuations. Overall, we have in the past therefore across the placode observed a spread of cycle lengths (Booth et al., 2014). This makes analysis more difficult especially in terms of combining individual cell analyses.

What we have done now rather than showing the previous individual static intensity profile comparisons from the original submission, is two-fold:
- we have followed trajectories of pulses across time for each combination of markers which shows matched patterns of apical-medial foci movement, supporting dynamic colocalization.
- we have quantified apical-medial intensity of the hub components in comparison to b-H-Spec (myo, Shot, Patronin) in a time-resolved way for individual cells (several cells per genotype from at least 3 different embryos each) and present examples of these in the main figure and more in the supplement. Superposition of these graphs is not possible as amplitude and cycle length vary quite significantly, but qualitatively these plots in our view show strong congruence in dynamic intensity fluctuations by b-H-Spec with SqhRFP as well as by b-H-Spec with Shot-EGFP. By contrast PatroninRFP does not fluctuate as much or at all in comparison to b-H-Spec, likely due to the fact that Patronin is bound and stabilises microtubule minus ends. This is now all shown in the revised version of Figure 2 and Supplemental Figure S2.
The combination of spatial analysis of foci movement over time (trajectories) plus time-resolved intensity fluctuations we feel supports our claim of overall matched dynamic behaviour between b-H-Spec, myosin, Shot and Patronin, either in space or time or both.

*****

Reviewer 2 Advance Summary and Potential Significance to Field:

This paper reports on studies on the regulation of tubular structure formation during morphogenesis using salivary gland development in Drosophila as a model system. In this system, locally orchestrated apical constriction in a defined region drives the invagination of epithelial cells and initiates tub formation. Apical constriction is a key mechanism of morphogenesis and studying its regulation is important. Using genetics and immunohistochemsity, quantitative live imaging and perturbations such as induced protein degradation and the use of dominant negative tools, the study reports the existence of a hub of proteins at the apical medial area of epithelial cells primed to invaginate. The authors identify β-H-Spectrin as a key component of this hub, that when acutely degraded leads to defects in apical constriction in the salivary gland context. The study further identifies that some components of the apical medial hub rely on microtubules for their localisation, while some including β-H-Spectrin and Big Bang do not. For β-H-Spectrin the authors gather evidence that its ability to interact with phospholipids of the apical plasma membrane perhaps enriched with P(3,4,5)P3 helps to position it at the apical medial area. Therefore, this

© 2024. Published by The Company of Biologists under the terms of the Creative Commons Attribution License (https://creativecommons.org/licenses/by/4.0/).

study provides novel insights into apical constriction regulation in this system with mechanistic insights into protein positioning and function.

The study is carried out overall at high standard. It brings novel mechanistic insights and even clarifies a long-standing observation why overexpressing spectrin's PH domain causes dominant negative effects during salivary gland development.
The manuscript could be improved however it its presentation and other aspects listed below before publication.

Reviewer 2 Comments for the Author:
1)The abstract could be rewritten to make a clearer distinction between what was known and what is new.

We have made this clearer in the revised version of the abstract.

2)The model presented in figure 7 is difficult to digest and should be revised. I enjoyed the insightful discussion. Illustrating conceptually what β-H-Spectrin might do could be better use for this figure.

We have updated and simplified Figure 7 and hope the reviewer will find it more intuitive and easier digestible.

3)It would be good to discuss why β-H-Spectrin at the junctions resists degradation, are there different pools with different functions?
We agree with the reviewer and strongly suspect that the apical-medial and junctional pools of β-H-Spectrin show a difference in accessibility. We have now added the below sentence to the results section on page 7:
'This differential response of apical-medial compared to junctional β-H-Spectrin-YFP to degradation could reflect a difference in accessibility due to different spatial arrangements of β-H-Spectrin and associated protein within both positions.'

We suspect this difference reflects a more general difference in arrangement between both positions for proteins that can be found in both. We have previously observed that when a dominant-negative version of Shot was expressed in the placode or when Patronin levels were reduced by RNAi approaches, that the apical-medial pools of these proteins were reduced and affected before the junctional pools were (Booth et al, 2014; Gillard et al., 2021). This could reflect a faster turnover in the apical-medial pool that might make it more susceptible to an overall reduction in levels.

4)The change of the apical area certainly also involves contractions at the junctions. How efficient is degradation of β-H-Spectrin? Its levels at the junctions appear reduced as well. Is the tension in the junctions the same? The lack of apical constriction could be caused by effects on both apical medial and junctional contractility.

The consensus within the morphogenesis field of groups studying apical constriction or cell wedging driven by apical-medial actomyosin in different tissues and organisms seems to be that the actual reduction of the apical area relies on the apical-medial pool of actomyosin, through pulling of this network on junctional contacts (and this pulling can be seen in time-lapse movies by the membrane deformation, we observe that within the salivary gland placode), but that junctional actomyosin is then required to stabilise a newly achieved shrunken state.
So both are important and play roles. The extent to which both pools are important is often difficult to dissect and they depend on many of the same regulators. During mesoderm invagination in Drosophila, both aspects appear controlled downstream of different transcription factors, with snail mutants affecting the contractions and twist mutants failing to stabilise a contracted state (Martin et al., 2009). As can be seen in the mesoderm and also the salivary gland placode, each cycle of medial myosin increase is closely followed by the reduction in apical area (Martin et al., 2009; Booth et al., 2014), whereas there is no such correlation with junctional myosin.

© 2024. Published by The Company of Biologists under the terms of the Creative Commons Attribution License (https://creativecommons.org/licenses/by/4.0/).

In fact we have shown that junctional myosin polarisation correlates with individual junction shrinkage of cells that are not apically constricting yet and are localised at a distance to the invagination point, where these circumferentially polarised myosin accumulation drive the initiation of cell intercalation events, pulling cells into T1 exchanges or multicellular rosette structures (Booth et al., 2014).
Coming back to the previously discussed point, there might well be a difference in accessibility of β-H-Spectrin-YFP to degradation in between apical-medial and junctional regions, leaving β-H-Spectrin-YFP at junctions more inaccessible, or the difference could be a more general difference in turnover that leads to faster depletion of apical-medial components in different experimental scenarios.

Testing tension through laser ablations in either wild-type or degradation situation is complicated by the fact that we know that (partly published, partly unpublished):
- there is a radial gradient of tension that develops across the placode once invagination begins
- radial versus circumferential junctions show differences in tension
So even if we cannot rule out potential changes in tension at junctions in β-H-Spectrin depleted embryos we feel the apical area quantifications that can be done in larger number provide a more reliable quantitative assessment of what is going wrong.

5)Figure 2, while I am inclined to follow the authors interpretation, could be more rigorously quantified. Could you analyse correlations, or better cross correlation and plot those? The areas chosen for line profiles seem a little arbitrary.

Please see our response to a very similar comment by Reviewer 1.

6)Figure 3 E clarify the sample size annotation.

For all quantitative graphs, the number of cells and number of independent embryos analysed is stated in the figure legend as well as in brackets underneath the analysed genotypes (i.e. 942c/24e meaning 942 cells from 24 embryos).

7)Figure 3, F, G, example placodes, where is the invagination pit? The white "bay" top rightish? Does this mean there are more cells with reduced apical area around the pit when Spectrin is degraded?

We apologise that this was not clear. We have now also here added asterisks to indicate the position of the pit, which was correctly identified by the reviewer as the 'white bay'. Cells directly in the pit due to the curvature the invagination generates at the tissue level have their apical area at an angle that makes proper segmentation not possible.
Within the region where cells begin to apically constrict and internalise, i.e. the dorsal-posterior region of the placode and an area slightly anterior to it (see e.g. Sanchez-Corrales et al. 2018), there is no finer grain stereotypic pattern of which cells will contract when. For this reason, we quantify cell area across the placode as presented in F-H' in many examples. Overall, when b-H-Spectrin is degraded, there are fewer constricting cells, but the area where cells constrict is not changed.
So no, it might just look like more constricted cells directly next to invagination pit in this example.

8)E.g. Figure 5. I might have misunderstood, but according to the methods quantifications as in Fig 4C require salivary gland fate visualisation via dCrebA. Both Cadherin and dCrebA are mouse? Clarify legends and quantification basis.
Counter-labeling for CrebA was used for the analysis displayed in Figure 3A-H', where placodes were analysed in three channels, with the first channel being bH-Spec-YFP (to be degraded), CrebA labelling to highlight placode nuclei, and E-Cadherin labelling to outline apical areas for the quantification.

In all other quantifications (Figures 3I-K, 4 and 5), we did not use CrebA to label salivary gland fate. The salivary gland placode area can be easily identified morphologically (aligned apical junctions at the boundary where a supracellular actomyosin cable forms, see Röper, 2021; Sidor et

© 2024. Published by The Company of Biologists under the terms of the Creative Commons Attribution License (https://creativecommons.org/licenses/by/4.0/).

al., 2020). In these experiments for Figures 3, 4 and 5, one channel is taken up in Figures 3 and 4 by the labelling for protein to be degraded (bH-Spec-YFP), channel 2 for the membrane label (E-Cad, to identify apical areas) and channel 3 for the analysed component (i.e. F-actin/phalloidin or acetyl. tub or Patronin or Shot). For Figure 5, channel 1 is for the bH-Spec or Bbg protein traps (YFP/GFP), channel 2 for acetyl. a-tubulin staining (because as we and others reported the spastin expression does not work in all cases, so actual loss of microtubules always has to be assessed), and channel 3 for apical membrane label (E-Cad) to be able to quantify per individual cell.
We did not routinely do quadruple labels.
The E-Cadherin antibody is rat mAb, for CrebA labelling both a rat mAb and a rabbit pAb exist, so the combination of E-Cad (rat) plus CrebA (rabbit) was used in Figure 3 A-H'.

We have revised the methods section to make the approaches clearer.

9)Figure 4, it would be a good idea to check that degradFP expression does not affect apical cell area by itself, that control is missing.

We have used interchangeably as control either the bH-Spec-YFP fkhGal4 or bH-Spec-YFP UAS-degradFP stocks, and both show no effect on salivary gland invagination or apical area change at all. The reviewer is right, we have not also tested just expressing UAS-degradFP under fkhGal4 control without any tagged transgene present that would be a target. The original paper describing the degradFP system in flies (Caussinus et al., 2012) states in its results: 'Ubiquitous expression of NSlmb-vhhGFP4 using tubulin-Gal4 (tubGal4) or nullo-Gal4 (nulloGal4) strong drivers did not affect fly development and produced healthy and fertile adults.'

Furthermore, combinations of UAS-degradFP with various Gal4 drivers are available from stock centres, again emphasising that UAS-degradFP expression itself is unlikely to have any effect during important processes such as apical constriction.

10)Parts of the discussion are too redundant (e.g. page 13 second paragraph lower part) with wording in the results.

We have deleted a repeated section (from the results section) and shortened this part.

11)A table with the genotypes for each figure could help and the figure legends could be improved to contain all necessary detail.

We have now added such table to Supplemental Materials (as no tables are permitted by JCS in the Methods section).

*****

Reviewer 3 Advance Summary and Potential Significance to Field:
This well-written and thorough paper is a welcome addition to the literature on the cytoskeletal basis for apical constriction, a crucial cell shape change that drives epithelial bending during development of many organs and organisms.

Building on the lab's own previous work using the Drosophila salivary gland, the authors describe several new protein components of the so-called "apical hub" that organizes the medial pool of actomyosin that is crucial for apical constriction. The core finding is that B-h-spectrin is present in the hub where it is essential for effective apical constriction. Loss of spectrin leads to loss of several hub components related to actin but does not perturb the organization of microtubules. A final set of structure/function analyses reveals that the spectrin PH domain is essential for its function here and accordingly that local control of phospholipids may be important for apical constriction.

Overall the paper is very convincing and I support publication after a few minor concerns are addressed.

Reviewer 3 Comments for the Author:

Comments/Concerns:

© 2024. Published by The Company of Biologists under the terms of the Creative Commons Attribution License (https://creativecommons.org/licenses/by/4.0/).

1. The section heading on page 7 states flatly that Spectrin "is required for apical constriction and assembly" of the hub, but all the effects shown in Fig, 3 are fairly modest. I think it would be more accurate to say that spectrin is required for "effective" or "complete" apical constriction, etc. Hedging here a little bit would not weaken the paper.

We are happy to tone this down and agree that it is not a black and white change and use the suggested term of 'effective'.

2. Two things in Fig 5A'' and B'' don't make sense to me. First, in the images shown, the apical hub as labeled by spectrin in green seems much larger in all cells expressing spastin, but the graph says there's no difference. The graph says "accumulation", so is this just mean pixel intensity? If so, it may not detect a change in hub size. Perhaps the authors should quantify area of the hub? Or perhaps they just picked a non-representative image to show in the figure. Either way this should be resolved.

As we show in Figure 2 and discuss, myosin and the other components of the apical-media hub including b-H-Spectrin undergoes fluctuations in localisation as well as intensity across apical constriction cycles.
We agree that qualitatively the foci in the particular image shown originally in B'' appeared slightly larger, but looking across the many images we collected this is not general feature, and is also what the quantification of many embryos and cells per embryo has shown and as presented, this shows quite clearly no overall difference in intensity across the apical-medial region.
We have replaced the panels in B-B'' with a more representative one.

Second, to my eye, the spastin expression also causes a robust ectopic accumulation of spectrin at the cell cortex. Is this the case? If so it should be quantified and discussed. Given the interplay of apical and medial actin populations, this could be an informative result.

We have assessed the junctional accumulation of control and experimental cases in all images quantified previously for the apical-medial accumulation of b-H-Spectrin.
This has in fact revealed that when Spastin is expressed junctional b-H-Spectrin appears slightly reduced (see below). It is unclear what the cause of this change is, but an explanation could be that some microtubule-interaction with junctions is required to stabilise other interactors of b-H-Spectrin at these junctions.
Although there is most likely some interaction/exchange/interdependence between components that show localisation to both junctions and the apical-medial hub, the mechanisms of recruitment and maintenance are likely distinct. Dissecting this interplay we feel would be a whole separate study.

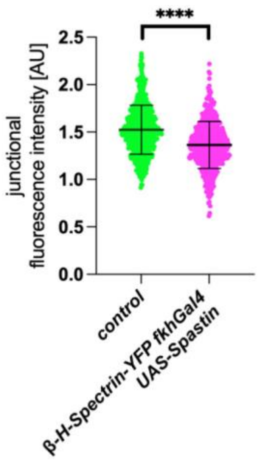

3. No arrows lead to or from Filamin in the right-hand panel of Fig. 7.

You are correct, we should have included a green arrow from actin to Filamin/cheerio. We have not addressed any further dependencies for Filamin for its recruitment or the effect of its loss on other components (as the tissue-specific degradation did not work efficiently enough).

© 2024. Published by The Company of Biologists under the terms of the Creative Commons Attribution License (https://creativecommons.org/licenses/by/4.0/).

4. The paper is quite scholarly, with a robust introduction and discussion.
But an opportunity is missed in these sections' very heavy focus on Drosophila, and occasionally C. elegans.
It would be useful to the community, I feel, for the authors to mention that microtubules, spectrins, and PH domains are all also implicated in apical constriction by studies in Xenopus. For example, parallel microtubule arrays with minus ends at the apical surface are a hallmark of apical constriction in Xenopus gastrulation (PMID: 17868669) and neurulation (PMID: 20534674; PMID: 17329357), and Shroom family proteins that control these microtubule arrays and have also been related to spectrin localization (PMID: 19554350). Finally, the PH-domain protein Plekhg5 controls apical constriction in Xenopus gastrulation (PMID: 30446627). These evolutionarily conserved aspects of apical constriction are important, especially because so many key regulators in Drosophila apical constriction (e.g. Fog pathway) seem largely not to be.

The reviewer is right to mention these studies that support that what we are analysing is a structure and process that appears conserved across evolution. We have added sections discussing these other instances to both introduction and discussion.

Second decision letter

MS ID#: JOCES/2024/261946

MS TITLE: ß-H-Spectrin is a key component of an apical-medial hub of proteins during cell wedging in tube morphogenesis

AUTHORS: Ghislain Gillard and Katja Röper

I am happy to tell you that your manuscript has been accepted for publication in Journal of Cell Science, pending standard publication integrity checks.

Reviewer 1

Advance summary and potential significance to field

The authors have nicely incorporated my suggestions into the revised version of their manuscript. As stated previously, this work will have impact on the field and will serve as a strong foundation for future studies. I feel that it is ready for publication.

Comments for the author

None.

Reviewer 2

Advance summary and potential significance to field

In this study the molecular mechanism driving apical constriction in the placode of the developing salivary glands in Drosophila is examined with a focus on the role of ß-H-Spectrin. The authors first convincingly show that Sqh, alpha-Shot, Patronin, ß-H-Spectrin, Filamin and Big Band all colocalise in what they call the 'hub' at the apical medial region where microtubules and the actin filaments intersect in the epithelial cells undergoing apical constriction. They then go on investigating the dynamics of these proteins and find that most follow Myosin II behaviour in this system. Based on their own findings and reports from the literature they then hypothesised that ß-H-Spectrin, based on its localisation dynamics, might prime the epithelial cells to undergo apical constriction which they tested using degradFP mediated depletion of it measuring the effect. ß-H-Spectrin was found

© 2024. Published by The Company of Biologists under the terms of the Creative Commons Attribution License (https://creativecommons.org/licenses/by/4.0/).

to remain on junctions, but the apical medial 'hub' localisation was gone and the localisation of the above identified components also changed and consequently apical constriction was less frequent in the system. They further provide evidence that ß-H-Spectrin's role in this process is different from that of Patronin's and that ß-H-Spectrin's localisation does not depend on microtubules for its localisation to the 'hub' region. Next they investigated how ß-H-Spectrin is localised. Based on ß-H-Spectrin's structure they speculate that binding to phosphoinositides might be key. They found indeed using characterised and established reporters for PI(3,4,5)P3 and PI(4,5)P2 that phosphoinositides cluster where the 'hub' and ß-H-Spectrin localise. To test if the phosphoinosite interaction motif on ß-H-Spectrin is functional relevant they then overexpressed the ß-H-33 fragment containing this motif to see if this can outcompete endogenous ß-H-Spectrin (using a mutant ß-H-33 fragment that cannot bind PIPs as a control) and found effects on ß-H-Spectrin localisation in the experiment but not the control. This affected both ß-H-Spectrin localisation and to some extend apical constriction.
These are interesting novel findings relevant to the general developmental biology and cell biology communities. Especially for the field of apical constriction and morphogenesis which is widely studied in invertebrates and vertebrates alike.

Comments for the author

The revised manuscript reads well. The authors have significantly improved the manuscript in terms of clarity data representation and interpretation. I have no further criticism and recommend publication.

Reviewer 3

Advance summary and potential significance to field

Same as previous review.

Comments for the author

My concerns have been addressed and i now support publication in JCS.

© 2024. Published by The Company of Biologists under the terms of the Creative Commons Attribution License (https://creativecommons.org/licenses/by/4.0/).
